# Relationship of Arterial Stiffness Index and Pulse Pressure With Cardiovascular Disease and Mortality

**DOI:** 10.1161/JAHA.117.007621

**Published:** 2018-01-22

**Authors:** M. Abdullah Said, Ruben N. Eppinga, Erik Lipsic, Niek Verweij, Pim van der Harst

**Affiliations:** ^1^ Department of Cardiology University Medical Center Groningen University of Groningen The Netherlands; ^2^ Durrer Center for Cardiogenetic Research Netherlands Heart Institute Utrecht The Netherlands

**Keywords:** arterial stiffness, cardiovascular disease, cardiovascular outcomes, mortality, pulse pressure, UK Biobank, Aging, Cardiovascular Disease, Risk Factors, Blood Pressure, Mortality/Survival

## Abstract

**Background:**

Vascular aging results in stiffer arteries and may have a role in the development of cardiovascular disease (CVD). Arterial stiffness index (ASI), measured by finger photoplethysmography, and pulse pressure (PP) are 2 independent vascular aging indices. We investigated whether ASI or PP predict new‐onset CVD and mortality in a large community‐based population.

**Methods and Results:**

We studied 169 613 UK Biobank participants (mean age 56.8 years; 45.8% males) who underwent ASI measurement and blood pressure measurement for PP calculation. Mean±SD ASI was 9.30±3.1 m/s and mean±SD PP was 50.98±13.2 mm Hg. During a median disease follow‐up of 2.8 years (interquartile range 1.4–4.0), 18 190 participants developed CVD, of which 1587 myocardial infarction (MI), 4326 coronary heart disease, 1192 heart failure, and 1319 stroke. During a median mortality follow‐up of 6.1 years (interquartile range 5.8–6.3), 3678 participants died, of which 1180 of CVD. Higher ASI was associated with increased risk of overall CVD (unadjusted hazard ratio 1.27; 95% confidence interval [CI], 1.25–1.28), myocardial infarction (1.38; 95% CI, 1.32–1.44), coronary heart disease (1.31; 95% CI, 1.27–1.34), and heart failure (1.31; 95% CI 1.24–1.37). ASI also predicted mortality (all‐cause, CVD, other). Higher PP was associated with overall CVD (1.57; 95% CI, 1.55–1.59), myocardial infarction (1.48; 95% CI, 1.42–1.54), coronary heart disease (1.47; 95% CI, 1.43–1.50), heart failure (1.47; 95% CI, 1.40–1.55), and CVD mortality (1.47; 95% CI, 1.40–1.55). PP improved risk reclassification of CVD in a non–laboratory‐based Framingham Risk Score by 5.4%, ASI by 2.3%.

**Conclusions:**

ASI and PP are independent predictors of CVD and mortality outcomes. Although both improved risk prediction for new‐onset disease, PP appears to have a larger clinical value than ASI.

**Clinical Perspective**

**What Is New?**

- Analyses of the largest arterial stiffness index data set (n=169 613) to date indicated that it was an independent predictor of incident cardiovascular disease and all‐cause mortality.
- Arterial stiffness index improved the 5.9‐year risk prediction model of incident cardiovascular events by 2.3% when added to the Framingham Risk Score.
- Pulse pressure improved the 5.9‐year risk prediction model by 5.4% when added to the Framingham Risk Score.

**What Are the Clinical Implications?**

- Pulse pressure appears to have more added value than arterial stiffness index to improve the risk classification of incident cardiovascular disease.

## Introduction

As the arterial system ages, the large elastic arteries undergo progressive luminal dilatation, thickening of the arterial wall, increased deposition of collagen, and combined fragmentation and degeneration of elastin.^1^ The result of these changes is stiffening of the arteries and consequent increase in pulse‐wave velocity (PWV), which is used to assess arterial stiffness. Increased arterial stiffness can cause isolated systolic hypertension, which increases pulse pressure (PP). Arterial stiffness and PP are independent measures of vascular aging.^2^ PP is strongly related to adverse outcomes such as coronary heart disease (CHD), and cardiovascular events in hypertensive patients,^3^ elderly,^4^ and the general population.^5^ Several studies have observed carotid‐femoral (aortic) PWV, which is considered the criterion standard of arterial stiffness, to be strongly related to risk factors such as atherosclerosis,^6^ hypertension,^7^ metabolic syndrome,^7^ diabetes mellitus,^7^ and future cardiovascular disease (CVD) events,^8^ including CHD, stroke,^8^ and all‐cause mortality.^8^ Arterial stiffness index (ASI) is a convenient and noninvasive method to measure arterial stiffness by using infrared light (photoplethysmography) to record the volume waveform of the blood in the finger. The shape of the waveform is directly related to the time it takes for the pulse wave to travel through the arterial tree. These tools might be of interest to quickly estimate CVD risk.^9^, ^10^

In this study we investigated the association of vascular aging as indicated by ASI and PP with CVD risk factors, CVD events, and mortality in 169 613 participants from UK Biobank.

## Methods

### UK Biobank Participants

The data from the UK Biobank resource are available for other researchers following an approved research proposal.^11^ The UK Biobank study design and population have been described in detail elsewhere.^12^ In brief, UK Biobank is a large community‐based prospective study in the United Kingdom that recruited >500 000 participants aged 40 to 69 years old with the aim of improving prevention, diagnosis, and treatment of a plethora of illnesses including cancer, diabetes mellitus, stroke, and heart diseases. A total of 190 077 participants had an ASI measurement during their first or 1 of the follow‐up visits. We analyzed data from 169 829 participants who had an ASI assessment at their first visit to the assessment centers in England and Wales. No ASI assessments were performed for participants from Scotland. All participants gave informed consent for the study via a touch‐screen interface that required agreement for all individual statements on the consent form as well as the participant's signature on an electronic pad.^13^ In this process all participants gave informed consent for data linkage as 1 statement requested consent for access to medical and other health‐related records, the long‐term storage and use of this and other information about the participants, also after incapacity or death, for health‐related research. The UK Biobank consent form is available at: http://www.ukbiobank.ac.uk/wp-content/uploads/2011/06/Consent_form.pdf. UK Biobank has approval from the institutional review boards, namely, the North West Multi‐centre Research Ethics Committee for the UK, from the National Information Governance Board for Health & Social Care for England and Wales, and from the Community Health Index Advisory Group for Scotland.^14^

### Ascertainment of ASI

PWV for ASI assessment was measured during the first visit to the assessment center using the PulseTrace PCA2 (CareFusion, San Diego, CA) (Field‐ID 21021) in 169 829 participants from 2009 until 2010. The PulseTrace PCA2 uses finger photoplethysmography to obtain the pulse waveform during a 10‐ to 15‐s measurement using an infrared sensor clipped to the end of the index finger. The measurement was repeated on a larger finger or on the thumb if less than two thirds of the waveform was visible on the display of the PulseTrace PCA2 device, or if the waveform did not stabilize within 1 minute after clipping the infrared sensor to the end of the index finger.^15^ The shape of the waveform is directly related to the time it takes for the pulse wave to travel through the arterial tree.^10^, ^15^ The standing height (shoeless) was measured using a Seca 202 height measure and was manually entered into the assessment center software by a UK Biobank staff member. The software immediately alerted the staff member when he/she entered impossible or implausible values and was asked to correct it.^13^ Height (meters) was divided by the time between the peaks of the pulse waveform to obtain the ASI in m/s.^15^ This method has been validated by comparing it with carotid‐femoral PWV in 3 independent studies that concluded both measurements are highly correlated and that ASI is a simple, inexpensive, rapid technique that requires no training and is operator independent.^9^, ^10^, ^16^

### Ascertainment of Cardiovascular Events

The prevalence and incidence of cardiovascular risk factors, conditions and events were captured through self‐reported data collected at the assessment center using a questionnaire and a verbal interview. Diagnoses were additionally captured using the “Spell and Episode” category from the Hospital Episode Statistics records. This category contains main and secondary diagnoses, coded according to the *International Classification of Diseases Ninth Revision* (*ICD‐9*) and *10th Revision* (*ICD‐10*),^17^ made during hospital inpatient stay. The main diagnosis is taken to be the main reason for the hospital admission, while secondary diagnoses are more often contributory or underlying conditions. Furthermore, we used surgical procedures that were recorded according to the Office of Population, Censuses and Surveys: Classification of interventions and Procedures, version 4 coding.^18^ We used both the main and secondary diagnoses for recording prevalent and incident risk factors, conditions, and events. Incidence cases based on self‐reported diagnoses during follow‐up visits were included only if there were no events recorded according to *ICD‐9*/*ICD‐10*/Office of Population, Censuses and Surveys: Classification of interventions and Procedures and only if the participant did not report this in the previous visit. Date of event was then defined as reported age of diagnosis (when available) or the median date between the visit of the first self‐reported diagnosis and the previous visit. Follow‐up for new‐onset CVD, myocardial infarction (MI), CHD, heart failure (HF), stroke, and death because of these new‐onset cardiovascular conditions was from inclusion until March 31, 2015 for participants from England and until February 28, 2015 for participants from Wales. Please see Table S1 for the definitions used.

### Ascertainment of Mortality

Participant follow‐up for mortality started at inclusion in the UK Biobank study and was censored on January 31, 2016 for all participants from England and Wales. The information about cause of death was obtained from the National Health Service Information Centre. Detailed information about the linkage procedure is available online at http://biobank.ctsu.ox.ac.uk/crystal/refer.cgi?id=115559.

### Blood Pressure Measurements and PP and Mean Arterial Pressure Calculation

Systolic and diastolic blood pressure (Field ID 4079, 4080) were measured twice at the assessment center using an automated blood pressure device (Omron 705 IT electronic blood pressure monitor; OMRON Healthcare Europe B.V. Kruisweg 577 2132 NA Hoofddorp), or manually (Field ID 93, 94) using a sphygmomanometer with an inflatable cuff in combination with a stethoscope if the blood pressure device failed to measure the blood pressure or if the largest inflatable cuff of the device did not fit around the participant's arm.^19^ All measurements were performed while the participant was sitting in a chair and were carried out by nurses trained in performing blood pressure measurements.^19^ During the first measurement nearly all participants (169 529) had blood pressure measurements using the automated blood pressure device. All had a second automated blood pressure measurement, except 153 individuals who did not have a second measurement. Manual sphygmomanometer blood pressure measurements during the first measurement were performed on 84 participants (0.05% of the total population). Of these, 11 participants had no second measurement and all others had a second manual blood pressure measurement. Multiple available measurements for 1 individual were averaged. PP was calculated by subtracting the (average) diastolic from the (average) systolic blood pressure value. Mean arterial pressure (MAP) was calculated by dividing the PP by 3 and adding this value to the diastolic blood pressure. The Omron 705 IT blood pressure monitor has satisfied the Association for the Advancement of Medical Instrumentation SP10 standard and has been validated according to the British Hypertension Society protocol, with an overall “A” grade for both systolic and diastolic blood pressure measurements.^20^ However, since automated devices tend to measure higher systolic blood pressures than manual sphygmomanometers, we adjusted both systolic and diastolic blood pressures that were measured using the automated device using algorithms by Stang et al.^21^ For systolic blood pressure we used the following algorithm: 3.3171+0.9201×level (systolic blood pressure in mm Hg)+6.0246×sex (male=1; female=0). For diastolic blood pressure we used: 14.5647+0.8092×level (diastolic blood pressure in mm Hg)+2.0108×sex (male=1; female=0). These adjusted blood pressure values were used for all calculations, including the PP and MAP calculations.

### Statistical Analysis

Data are expressed as median (interquartile range) or as mean±SD for quantitative variables and as counts with percentages for discrete and categorical variables. Participants with ASI values below or above 4 SDs of the mean were excluded, as well as participants with incorrect inclusion dates and those younger than 40 years old, as this last group was considered less likely to undergo an event during follow‐up.

To evaluate whether ASI and PP increased with age, we performed regression spline models with 95% confidence intervals per 5 years of age for females and males separately.

We examined the effect of traditional cardiovascular risk factors on ASI and PP using unadjusted linear regression analyses and found all risk factors (sex, age, body mass index [BMI], MAP, diabetes mellitus, and smoking) were associated with both measures. We considered these risk factors in multivariable Cox regression models. We used up to 4 summative models. Model 1: Unadjusted; Model 2: Adjusted for age and sex; Model 3: Model 2+MAP, diabetes mellitus, smoking, and BMI; Model 4: Model 3+history of CVD, MI, CHD, HF, and stroke. To examine the predictive value of ASI and PP for new‐onset CVD, MI, CHD, HF, and stroke, we performed Cox regression analyses using Model 1 to 3. Participants with a history of CVD, MI, CHD, HF, or stroke, were excluded from the respective analyses. To examine the relationship between ASI and PP with all‐cause, CVD, and non‐CVD mortality, we applied Model 1 to 4. For the Cox regression analyses, we reported hazard ratios and 95% confidence intervals.

Kaplan–Meier failure curves were plotted for outcomes associated with ASI or PP. Because older individuals tend to have more CVD events and die sooner, the use of single ASI and PP distributions for the entire study sample would result in a greater proportion of older participants to undergo events, in comparison to the smaller proportion of younger participants with fewer events. To improve the balance of these proportions, we stratified for deciles of age at ASI and PP measurement. Age decile cut points for men were at 44, 49, 52, 56, 59, 61, 63, 65, and 67 years. For women the cut points were at 44, 48, 52, 55, 58, 60, 62, 64, and 67 years. We then determined ASI and PP distributions for each decile, separately for ASI and PP and by sex. Participants with ASI and PP measurements below and above the median of their respective sex‐ and age‐specific deciles were pooled together and compared. Log‐rank testing was performed to estimate the statistical difference between the medians.

Harrell's C‐indices, a generalization of the area under the receiver operating characteristics curve for data from Cox regression analyses, were computed for Model 3 for disease and Model 4 for mortality outcomes, both with and without additional adjustment for ASI or PP. Postestimation analysis was used to determine whether ASI or PP independently predicted outcomes compared with the traditional risk factors. We further investigated the possible clinical impact of ASI and PP measurements in UK Biobank by performing reclassification analyses using the net reclassification improvement (NRI) and the integrated discrimination improvement.^22^ For the reclassification analyses, we used a non–laboratory‐based Framingham Risk Score (FRS) in which BMI is used instead of cholesterol.^23^ Because the FRS calculates 10‐year risk and we had a maximum of 5.92 years of follow‐up, we divided the FRS risk estimates by 1.69 to represent the 5.92‐year risk. Individuals were classified into <5%, 5% to 15%, and >15% risk categories. *P*<0.05 was considered statistically significant. All analyses were performed using Stata version 14 (StataCorp. 2015; Stata Statistical Software: Release 14. College Station, TX: StataCorp LP).

## Results

### UK Biobank Participants

We studied 169 613 individuals (45.8% males; average age 56.8 years old) participating in UK Biobank and of whom PP and ASI had been measured. The sample selection strategy is presented in Figure S1 and baseline characteristics are presented in Table 1. In total, 333 042 UK Biobank participants were excluded from the analyses. Baseline characteristics of the excluded participants are shown in Table S2. Ethnic backgrounds of included and excluded participants (both >90% white) are provided in Table S2. The most common risk factor was past or current smoking, followed by hypertension. CHD was diagnosed most often (n=4326), followed by MI (n=1587), stroke (n=1319), and HF (n=1192). The overall mean ASI was 9.30±3.1 m/s and mean PP was 50.98±13.2 mm Hg. ASI and PP were weakly correlated with each other (*R* ^2^=0.01; *P*<0.001). Both increased with advancing age (*R* ^2^=0.04 for ASI; *R* ^2^=0.17 for PP; both *P*<0.001, Figure S2). Multiple cardiovascular risk factors (increased BMI, hypertension, MAP, diabetes mellitus, and smoking) were associated with ASI and PP (Table 1). Heart rate was positively associated with ASI and negatively associated with PP (Table 1).

**Table 1:** Baseline Characteristics of the Study Population (n=169 613) and Unadjusted Associations With ASI and PP

Characteristics	Mean±SD or n (%)	ASI		PP	
		β (95% CI)	*P* Value	β (95% CI)	*P* Value
Males	77 708 (45.8)	0.39 (0.38–0.40)	<0.001	0.46 (0.45–0.47)	<0.001
Age, y	56.77±8.16	0.03 (0.03–0.03)	<0.001	0.05 (0.05–0.05)	<0.001
Heart rate, bpm^a^	68.72±11.01	0.08 (0.08–0.08)	<0.001	−0.05 (−0.06 to −0.05)	<0.001
Body mass index, kg/m^2^ a	27.46±4.82	0.24 (0.23–0.25)	<0.001	0.18 (0.17–0.19)	<0.001
Blood pressure, mm Hg^a^					
Systolic	132.92±17.78	0.10 (0.10–0.10)	<0.001	0.50 (0.50–0.50)	<0.001
Diastolic	81.94±8.37	0.24 (0.24–0.25)	<0.001	0.39 (0.39–0.40)	<0.001
Mean arterial	98.94±10.65	0.19 (0.19–0.20)	<0.001	0.63 (0.63–0.63	<0.001
Hypertension	52 885 (31.2)	0.23 (0.22–0.24)	<0.001	0.56 (0.55–0.57)	<0.001
Diabetes mellitus	10 267 (6.1)	0.19 (0.17–0.21)	<0.001	0.31 (0.29–0.33)	<0.001
Hyperlipidemia	34 723 (20.5)	0.23 (0.22–0.24)	<0.001	0.40 (0.39–0.41)	<0.001
Past or current smoker	100 590 (59.3)	0.14 (0.13–0.15)	<0.001	0.06 (0.05–0.07)	<0.001

Means with SD or counts with percentages are given per characteristic. The β coefficients with 95% confidence interval (CI) are estimated using ASI per SD change in m/s and PP per SD change in mm Hg. The β coefficients show the extent to which the baseline characteristics have an effect on ASI and PP. ASI indicates arterial stiffness index; bpm, beats per minute; PP, pulse pressure.

a The β coefficients for heart rate, body mass index, and blood pressure are given per 10 units change, respectively.

### ASI and PP Are Associated With New‐Onset Cardiovascular Events

The median follow‐up duration for new‐onset disease events was 2.8 years (interquartile range 1.4–4.0). Kaplan–Meier failure curves for CVD divided by the median ASI and PP are shown in Figure. Curves for MI, CHD, and HF divided by the median ASI are shown in Figure S3 and curves for MI, CHD, and HF divided by the median PP in Figure S4. Log‐Rank testing showed a difference between below and above the median ASI and PP for most diseases. HF showed no difference between below and above the median ASI. In total, during a maximum of 5.9 years of follow‐up, CVD was diagnosed in 18 190 individuals. Cox regression analyses showed that both increased ASI and PP were associated with increased risk for CVD, MI, CHD, HF, and stroke (Table 2). Adjustment for age and sex did not alter the associations (Table 2). Additional adjustment for CVD risk factors (MAP, diabetes mellitus, smoking, and BMI) resulted in loss of the associations between ASI and PP with stroke and lead to small effect sizes for most outcomes (Table 2). Additional adjustment for PP did not affect the associations between ASI with the disease outcomes, nor did additional adjustment for ASI affect the association between PP with disease outcomes (Table S3). We also plotted the risk of quintiles of ASI and PP for overall CVD adjusted for the third model and observed semilinear increases in risk (Figure S5).

**Figure 1:**
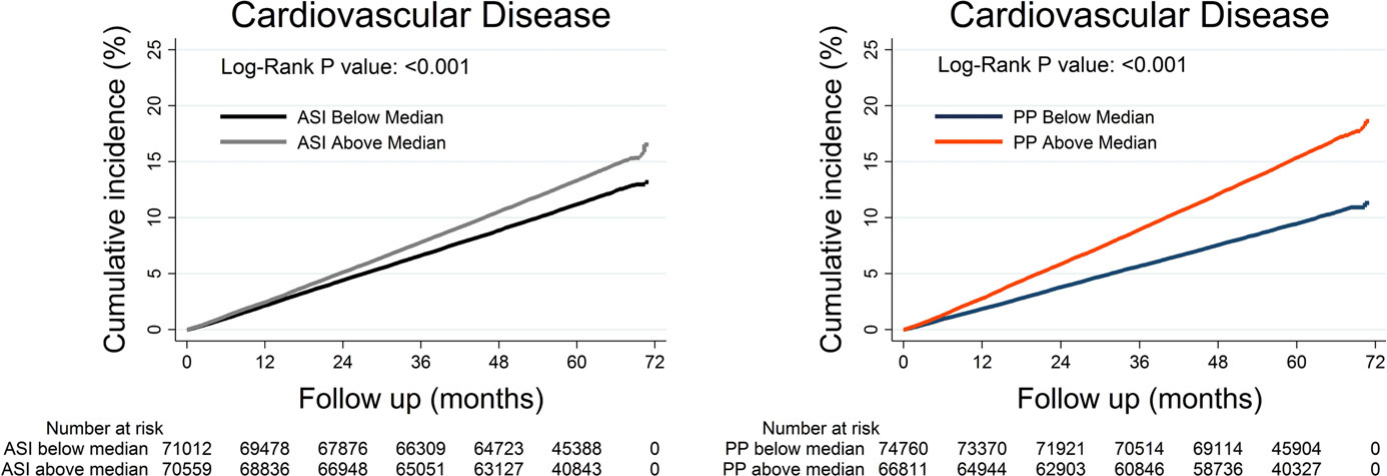
Shown are cumulative new‐onset cardiovascular disease in (%) divided by the median ASI (A) and the median PP (B). All curves were adjusted for age and sex. Log‐Rank testing shows significant differences for A and B. ASI indicates arterial stiffness index; PP, pulse pressure.

**Table 2:** Association of ASI and PP With New‐Onset Cardiovascular Diseases

	CVD n_total_=141 571 n_event_=18 190 (12.8%)		Myocardial Infarction n_total_=165 589 n_event_=1587 (1.0%)		CHD n_total_=162 543 n_event_=4326 (2.7%)	
	Hazard Ratio (95% CI)	*P* Value	Hazard Ratio (95% CI)	*P* Value	Hazard Ratio (95% CI)	*P* Value
ASI						
Model 1	1.27 (1.25–1.28)	<0.001	1.38 (1.32–1.44)	<0.001	1.31 (1.27–1.34)	<0.001
Model 2	1.11 (1.10–1.13)	<0.001	1.17 (1.11–1.22)	<0.001	1.11 (1.08–1.14)	<0.001
Model 3	1.04 (1.03–1.06)	<0.001	1.13 (1.07–1.18)	<0.001	1.08 (1.05–1.11)	<0.001
PP						
Model 1	1.57 (1.55–1.59)	<0.001	1.48 (1.42–1.54)	<0.001	1.47 (1.43–1.50)	<0.001
Model 2	1.32 (1.30–1.34)	<0.001	1.17 (1.11–1.23)	<0.001	1.15 (1.12–1.19)	<0.001
Model 3	1.05 (1.03–1.07)	<0.001	1.11 (1.04–1.19)	0.001	1.14 (1.09–1.18)	<0.001

	Heart Failure n_total_=168 751 n_event_=1192 (0.7%)		Stroke n_total_=166 954 n_event_=1319 (0.8%)	
	Hazard Ratio (95% CI)	*P* Value	Hazard Ratio (95% CI)	*P* Value
ASI				
Model 1	1.31 (1.24–1.37)	<0.001	1.25 (1.19–1.32)	<0.001
Model 2	1.09 (1.03–1.15)	<0.01	1.08 (1.02–1.14)	<0.01
Model 3	1.07 (1.01–1.13)	0.02	1.05 (1.00–1.11)	0.07
PP				
Model 1	1.47 (1.40–1.55)	<0.001	1.47 (1.40–1.54)	<0.001
Model 2	1.10 (1.04–1.16)	<0.01	1.16 (1.10–1.22)	<0.001
Model 3	1.21 (1.13–1.30)	<0.001	1.05 (0.98–1.13)	0.17

Hazard ratios (HR) with 95% confidence interval (CI) estimated using ASI per SD change in m/s and PP per SD change in mm Hg are shown per model for incident CVD, myocardial infarction, coronary heart disease, heart failure, and stroke. Model 1: univariate (unadjusted). Model 2: adjusted for age and sex. Model 3: Model 2+mean arterial pressure, diabetes mellitus, smoking, and body mass index. ASI indicates arterial stiffness index; CHD, coronary heart disease; CVD, cardiovascular disease; PP, pulse pressure.

Addition of ASI to traditional risk factors increased C‐indices for MI and CHD, independent of additional adjustment for PP (Table S4). Addition of PP to traditional risk factors increased C‐indices for CVD and CHD independently of adjustment for ASI, but it did not increase C‐indices for MI or stroke. The NRI showed improvement (2.3%; 95% confidence interval, 2.0–2.6; *P*<0.001) in reclassification when ASI was added to the FRS (Table 3). Also the integrated discrimination improvement was improved with an estimate of 0.0008 (*P*<0.001). When PP was added to the FRS, the NRI showed an improvement of 5.4% (95% confidence interval, 4.9–5.8; *P*<0.001; Table 4) and the integrated discrimination improvement was estimated at 0.006 (*P*<0.001). We calculated the cutoff values using Youden's index (indicating the value at which the measure has highest sensitivity and specificity) of ASI and PP for overall CVD at 9.21 m/s and 51.23 mm Hg, respectively.

**Table 3:** Reclassification of Predicted 5.9 Years Risk of New‐Onset CVD Events With the Addition of Arterial Stiffness Index to a Non–Laboratory‐Based Framingham Risk Score

Model Without ASI	Model With ASI				Risk Reclassification	
	<5%	5% to 15%	≥15%	Total	Higher	Lower
<5%						
Persons included	14 376	1536	0	15 912		
No. of events	352	68	0	420	68	NA
No. of nonevents	14 024	1468	0	15 492	1468	NA
5%–15%						
Persons included	3450	70 618	937	75 005		
No. of events	148	6673	205	7026	205	148
No. of nonevents	3302	63 945	732	67 979	732	3302
≥15%						
Persons included	0	2600	48 054	50 654		
No. of events	0	224	10 520	10 744	NA	224
No. of nonevents	0	2376	37 534	39 910	NA	2376
Total						
Persons included	17 826	74 754	48 991	141 571		
No. of events	500	6965	10 725	18 190	273	372
No. of nonevents	17 326	67 789	38 266	123 381	2200	5678
NRI	2.3% (95% CI, 2.0–2.6; *P*<0.001)					

ASI indicates arterial stiffness index; CI, confidence interval; CVD, cardiovascular disease; NA, not applicable; NRI, net reclassification improvement.

**Table 4:** Reclassification of Predicted 5.9‐Year Risk of New‐Onset CVD Events With the Addition of PP to a Non–Laboratory‐Based Framingham Risk Score

Model Without PP	Model With PP				Risk Reclassification	
	<5%	5% to 15%	≥15%	Total	Higher	Lower
<5%						
Persons included	14 588	1324	0	15 912		
No. of events	376	44	0	420	44	NA
No. of nonevents	14 212	1280	0	15 492	1280	NA
5%–15%						
Persons included	7198	64 609	3198	75 005		
No. of events	305	6055	666	7026	666	305
No. of nonevents	6893	58 554	2532	67 979	2532	6893
≥15%						
Persons included	0	4507	46 147	50 654		
No. of events	0	477	10 267	10 744	NA	477
No. of nonevents	0	4030	35 880	39 910	NA	4030
Total						
Persons included	21 786	70 440	49 345	141 571		
No. of events	681	6576	10 933	18 190	710	782
No. of nonevents	21 105	63 864	38 412	123 381	3812	10 923
NRI	5.4% (95% CI: 4.9–5.8; *P*<0.001)					

CI indicates confidence interval; CVD, cardiovascular disease; NA, not applicable; NRI, net reclassification improvement; PP, pulse pressure.

### ASI and PP are Associated With Mortality

Median follow‐up for mortality was 6.1 years (interquartile range 5.8–6.3). In total, 3678 participants died, of which 1180 participants of CVD. Kaplan–Meier failure curves for all‐cause, CVD, and non‐CVD mortality divided by the age‐ and sex‐specific median ASI are shown in Figure S6 and a curve for CVD mortality divided by the median PP in Figure S7. Log‐rank testing showed a difference for all mortality outcomes, except CVD mortality, which showed no difference between the ASI values. In univariate Cox regressions we observed that higher ASI and PP were associated with increased risk for all mortality outcomes (Table 5). The association between ASI with increased risk of all mortality outcomes persisted when adjusted for traditional risk factors and history of CVD, MI, CHD, HF, or stroke (Table 5). To investigate whether the association between ASI and non‐CVD mortality was driven by cancer mortality or other causes, we divided non‐CVD mortality into cancer mortality and death by other causes (non‐CVD/noncancer). ASI was associated with both cancer mortality and non‐CVD/noncancer mortality, also after adjustment for all factors mentioned above (Table S5). The associations of PP with all‐cause and non‐CVD mortality were less strong (Table 5).

**Table 5:** Association of ASI and PP With All‐Cause, CVD, and Non‐CVD Mortality

	All‐Cause Mortality n_total_=169 613 n_event_=3678 (2.2%)		CVD Mortality n_total_=169 613 n_event_=1180 (0.7%)		Non‐CVD Mortality n_total_=169 613 n_event_=2498 (1.5%)	
	Hazard Ratio (95% CI)	*P* Value	Hazard Ratio (95% CI)	*P* Value	Hazard Ratio (95% CI)	*P* Value
ASI						
Model 1	1.26 (1.23–1.30)	<0.001	1.35 (1.28–1.42)	<0.001	1.22 (1.18–1.27)	<0.001
Model 2	1.09 (1.05–1.12)	<0.001	1.11 (1.05–1.18)	<0.001	1.07 (1.03–1.12)	<0.001
Model 3	1.08 (1.04–1.11)	<0.001	1.10 (1.04–1.16)	0.001	1.07 (1.03–1.11)	<0.001
Model 4	1.08 (1.05–1.12)	<0.001	1.11 (1.05–1.17)	<0.001	1.07 (1.03–1.11)	<0.001
PP						
Model 1	1.32 (1.28–1.36)	<0.001	1.47 (1.40–1.55)	<0.001	1.25 (1.21–1.30)	<0.001
Model 2	1.01 (0.97–1.04)	0.66	1.08 (1.02–1.14)	0.01	0.98 (0.94–1.02)	0.25
Model 3	1.05 (1.00–1.09)	0.04	1.15 (1.06–1.23)	<0.001	1.00 (0.95–1.05)	0.94
Model 4	1.03 (0.99–1.08)	0.17	1.10 (1.02–1.18)	0.02	1.00 (0.95–1.05)	0.94

Hazard ratios with 95% confidence interval (CI) estimated using ASI per SD change in m/s and PP per SD change in mm Hg are shown per model for all‐cause, CVD, and non‐CVD mortality. Model 1: univariate (unadjusted). Model 2: adjusted for age and sex. Model 3: Model 2+mean arterial pressure, diabetes mellitus, smoking, and body mass index. Model 4: Model 3+history of CVD, myocardial infarction, coronary heart disease, heart failure, and stroke. ASI indicates arterial stiffness index; CVD, cardiovascular disease; PP, pulse pressure.

To determine whether ASI is a predictor for all‐cause, CVD, and non‐CVD mortality independently of PP, we adjusted the largest significant ASI Cox regression models for PP, which did not affect any of the associations (Table S6). Similarly, adjusting the PP Cox regression models for ASI did not affect the PP associations either. Postestimation C‐indices for all‐cause mortality were improved after ASI was added to the traditional risk factors and history of disease, independent of adjustment for PP (Table S4). C‐indices for CVD mortality did not improve when PP was added to the traditional risk factors and history of disease (Table S4).

In the literature there is ongoing debate whether PP^24^ and especially aortic PWV^25^, ^26^ measurements should be adjusted for heart rate. For this reason we performed sensitivity analyses adjusting the largest significant PP and ASI models for heart rate, but this did not affect our conclusions (Table S7).

## Discussion

We studied 2 independent measures of vascular aging,^2^ ASI and PP, in relation to CVD events and mortality in 169 613 participants of UK Biobank. In the largest sample size available to date, we demonstrated that ASI was an independent predictor of new‐onset CVD outcomes and mortality. Furthermore, we have shown that PP was an independent predictor of CVD, MI, CHD, HF, and CVD mortality. PP improved C‐indices of CVD and CHD, but not of MI, stroke, and CVD mortality, compared with traditional risk factors. PP added to the nonlaboratory FRS improved the NRI twice as much compared with ASI.

### Associations With CVD

We found that both increased ASI and PP were associated with an increased risk of developing CVD. The predictive value of ASI and PP can be considered a reflection marking the (patho)physiological function of the cardiovascular system. Stiffer arteries allow the pressure wave in the arterial tree to travel faster, causing systolic rather than diastolic augmentation of the reflected pressure wave. Systolic augmentation increases PP and left ventricular load, which in turn can lead to cardiac hypertrophy.^27^ In addition, ischemia of the left ventricle could also occur through decreased aortic pressure during diastole causing reduced coronary filling and thereby reduced myocardial perfusion.^27^, ^28^

In our study we found a positive association between overall CVD events and ASI whereby the risk increased with 6% per SD change. This association is in line with previous, smaller observations.^8^, ^29^ Both reported a higher risk (6.15% versus 1.6% risk per year) of developing CVD per SD increase in aortic PWV. This difference in effect size might be attributable to differences in baseline characteristics. For example, participants of the study by Mitchell et al^29^ were recruited from 1998 to 2001 and were on average 6.2 years older. The participants of the studies in the meta‐analysis of Vlachopoulos et al^8^ were recruited from 1980 to 2005 and were on average 2.4 years older than our participants.

The association between PP and CVD incidence was previously reported by Blacher et al^30^ in older (average 67–72 years) hypertensive patients who had a 17% increased risk of CVD per 10 mm Hg higher PP. The risk of developing CVD in this cohort of hypertensive subjects is higher than the risk observed in our presumably healthier community‐based population, which was 3.8% when estimated per 10 mm Hg higher PP (1 SD in our population equals 13.2 mm Hg difference in PP). As noted before, ASI and PP are independent measures of vascular aging; also in our study the correlation between ASI and PP was weak. ASI and PP likely do not reflect the same properties or characteristics of the vasculature. However, improvement in NRI was observed when ASI and PP were added to the non–laboratory‐based FRS, indicating both ASI and PP may aid the risk prediction of overall CVD. The improvement of the NRI by addition of ASI was low (2.3%), whereas the improvement of the NRI by addition of PP was much larger (5.4%), indicating a superior possible clinical applicability of PP compared with ASI. The NRI possibly better reflects the added clinical value of ASI and PP to the risk prediction than the C‐indices, which for each end point were similar until the third decimal. We also performed sensitivity analyses in subgroups and found PP was modestly better than ASI at predicting mainly overall CVD in, for example, women, participants with diabetes mellitus, and both young and old participants (Tables S8 through S12). ASI was better at predicting overall CVD in men and all‐cause mortality in women, nondiabetics, and young participants (≤56.8 years). ASI and PP were not different in subgroups of hypertension and FRS (Tables S9 and S11). However, also in these additional analyses the C‐indices were very similar. The significant differences in C‐indices between different models of the main‐ and subgroup analyses may be attributable to the large sample size and does not represent a difference that is clinically relevant.

Besides finding an association between ASI and PP with overall CVD, we observed associations between ASI and PP with several specific disease outcomes. Both ASI and PP were associated with MI and CHD in our community‐based population. In aortic PWV studies, MI was often included in the definition of outcome variables such as overall CVD events or CHD, but not investigated as an individual outcome as has been done in this study. These previous studies show an association between aortic PWV and CHD,^8^, ^31^, ^32^, ^33^ as is shown here as well. Our results are consistent with previous PP studies that reported associations with both incident MI^3^ and CHD^34^ after adjustment for traditional CVD risk factors in a hypertensive and community‐based cohort, respectively.

The association between aortic PWV with HF is inconsistent across studies.^32^, ^35^ We found an association between ASI and HF after adjustment for traditional CVD risk factor but it had little discriminating power. We found a strong association between PP and HF, similar to 2 previous studies that found PP independently predicted chronic HF in the Framingham Heart Study population^36^ and an elderly population.^37^

Finally, neither ASI nor PP was associated with stroke in our population in survival models after adjustment for MAP. The association between arterial stiffness measured by carotid‐femoral PWV index and stroke, which was also adjusted for MAP, has, however, been described previously.^31^ Also the associations of PP with stroke are inconsistent with earlier work in older people with isolated systolic hypertension, where they found an 11% increased risk per 10 mm Hg higher PP, but also showed that MAP increased risk of stroke.^38^ However, a later study in uncontrolled hypertensive subjects found, similar to this study, that the relationship between PP and stroke was dependent on MAP,^39^ indicating that MAP may be a confounder in the association between PP and stroke.

### Associations With Mortality

In the present work we found that ASI was associated with all‐cause, CVD, and non‐CVD mortality, although no statistical difference between CVD mortality incidences was found between individuals with high and low ASI values. The association between ASI and non‐CVD mortality appears to be driven by both cancer mortality and mortality by other causes. To our knowledge, increased ASI has not previously been found to predict cancer mortality. The origin of this association should be subject to future research. Unlike ASI, and in contrast to previous reports of studies in 9431 (65–102 years old)^40^ and 2725 (20–80 years old)^41^ individuals from the general population, PP was not convincingly associated with all‐cause or non‐CVD mortality.

### Heart Rate

The addition of heart rate to the ASI models had little effect, suggesting that heart rate had no confounding effect on ASI, unlike previously suggested for aortic PWV.^25^, ^26^ The addition of heart rate to the PP models had similar little effect, arguing against a need for heart rate adjustment for PP.^24^ It is interesting that heart rate was inversely associated with PP, whereas it was positively associated with ASI. However, it should be noted that the effect of heart rate is small for both measures, also when compared with the effects of the other characteristics in Table 1.

### Strengths and Limitations

The associations between ASI and PP with disease and mortality outcomes have been studied previously but our contemporary study is unique. Not only is the very large sample size unprecedented, but also we provide a community‐based population with both ASI and PP measurements in combination with detailed health‐ and mortality‐related data. Although the effect sizes were small to moderate for most outcomes in the unadjusted Cox regression analyses and even smaller in the adjusted analyses, the large sample size allowed us to detect these at the statistically significant level. One important limitation is the limited duration of disease follow‐up. A second limitation is that, although we could adjust for a number of important classical risk factors, we did not have data available on serum lipid levels to take into account in our multivariable models. A third limitation is that the accuracy of Hospital Episode Statistics data used for our analyses is not known for most data fields. Furthermore, the methodological differences of arterial stiffness measurement (aortic PWV versus ASI) are likely to play a role in the discrepancies found between our and previous studies. ASI derived by finger photoplethysmography is influenced by the elasticity of the large central arteries and the properties of the reflection sites, both central and peripheral.^16^, ^25^ In addition, the PWV measured in the peripheral arteries are usually higher than the aortic PWV because of the nearer reflection sites.^25^ These differences make results from our and previous studies (aortic PWV) more difficult to directly compare. Finally, brachial PP was used as a measure for systemic arterial stiffness instead of central PP, which is measured at the carotid artery. Brachial PP is generally higher than central PP because of the larger number of reflection sites in the peripheral arteries compared with the central arteries. The central arteries of younger individuals are more elastic than the peripheral arteries, which can further increase difference between brachial PP and central PP, and might have resulted in an overestimation of the stiffness of their arterial tree.^7^

### Perspectives

Higher ASI is associated with cardiovascular risk factors and is an independent predictor of new‐onset CVD outcomes as well as all‐cause, CVD, and non‐CVD mortality. Although finger photoplethysmography is a simple and fast method, ASI measurement added relatively little to the risk prediction in this community‐based population, limiting its potential clinical value. Similar to ASI, PP was an independent predictor of new‐onset CVD outcomes and CVD mortality. PP improved the CVD risk prediction classification by 5.4%, suggesting that PP could be used as a clinical tool to improve risk prediction for disease outcomes in patients. Also similar to ASI, PP is obtained with minimal efforts, further favoring the use of PP over ASI in clinical settings. ASI may, however, remain an interesting measure for studying vascular aging and stiffness.^42^

## Sources of Funding

Said was supported by the Royal Netherlands Academy of Arts and Sciences’ Van Walree grant. Verweij is supported by Marie Sklodowska‐Curie GF (call: H2020‐MSCA‐IF‐2014, Project ID: 661395) and an NWO VENI grant (016.186.125).

## Disclosures

None.

## Supporting information


**Table S1.** Variable Definitions Used in UK Biobank
**Table S2.** Baseline Characteristics of Included and Excluded UK Biobank Participants
**Table S3.** Independent Association of ASI and PP With Disease
**Table S4.** Harrell's C‐Indices and Postestimation Analyses of Models for Risk Prediction of Disease and Mortality With and Without ASI or PP
**Table S5.** Association of ASI With Non‐CVD/Noncancer and Non‐CVD Cancer Mortality
**Table S6.** Independent Association of ASI and PP With Mortality
**Table S7.** Association of ASI and PP With Diseases and Mortality, Additionally Adjusted for Heart Rate
**Table S8.** Harrell's C‐Indices for ASI and PP in Diabetics and Nondiabetics
**Table S9.** Harrell's C‐Indices for ASI and PP in Participants With and Without Hypertension
**Table S10.** Harrell's C‐Indices for ASI and PP in Participants Younger or Older Than the Median 56.8 Years
**Table S11.** Harrell's C‐Indices for ASI and PP in Participants With Lower or Higher FRS Than the Median
**Table S12.** Harrell's C‐Indices for ASI and PP in Men and Women Separately
**Figure S1.** Shown are the inclusion and exclusion criteria for the sample selection strategy used in our analyses. ASI indicates arterial stiffness index; PP, pulse pressure.
**Figure S2.** Shown are regression spline models in which the red line with gray‐colored 95% confidence intervals (CI) represents the linear regression of ASI and pulse pressure over increasing age (per 5 years) for men and women separately. ASI indicates arterial stiffness index.
**Figure S3.** Shown are cumulative new‐onset myocardial infarction, coronary heart disease, heart failure, and stroke in (%) divided by the median ASI. All curves were adjusted for age and sex. Log‐Rank testing shows significant differences for both myocardial infarction and coronary heart disease. ASI indicates arterial stiffness index.
**Figure S4.** Shown are cumulative new‐onset myocardial infarction, coronary heart disease, and stroke in (%) divided by the median PP. All curves were adjusted for age and sex. Log‐Rank testing shows significant differences for all. PP indicates pulse pressure.
**Figure S5.** Shown are risk of overall cardiovascular disease for quintiles of arterial stiffness index and pulse pressure separately. The analyses were adjusted for age, sex, mean arterial pressure, diabetes mellitus, body mass index, and smoking. ASI indicates arterial stiffness index; PP, pulse pressure.
**Figure S6.** Shown are cumulative all‐cause, CVD, and non‐CVD mortality in (%) divided by the median ASI. All curves were adjusted for age and sex. Log‐Rank testing shows significant differences for all‐cause and non‐CVD, but not CVD mortality. ASI indicates arterial stiffness index; CVD, cardiovascular disease.
**Figure S7.** Shown is the cumulative CVD mortality in (%) divided by the median PP. The curve was adjusted for age and sex. Log‐Rank testing shows a significant difference. CVD indicates cardiovascular disease; PP, pulse pressure.

## References

[1] Lakatta EG . Arterial and cardiac aging: major shareholders in cardiovascular disease enterprises: Part III: cellular and molecular clues to heart and arterial aging. Circulation. 2003;107:490–497. 12551876 10.1161/01.cir.0000048894.99865.02

[2] Benetos A , Okuda K , Lajemi M , Kimura M , Thomas F , Skurnick J , Labat C , Bean K , Aviv A . Telomere length as an indicator of biological aging: the gender effect and relation with pulse pressure and pulse wave velocity. Hypertension. 2001;37:381–385. 11230304 10.1161/01.hyp.37.2.381

[3] Madhavan S , Ooi WL , Cohen H , Alderman MH . Relation of pulse pressure and blood pressure reduction to the incidence of myocardial infarction. Hypertension. 1994;23:395–401. 8125567 10.1161/01.hyp.23.3.395

[4] Vaccarino V , Holford TR , Krumholz HM . Pulse pressure and risk for myocardial infarction and heart failure in the elderly. J Am Coll Cardiol. 2000;36:130–138. 10898424 10.1016/s0735-1097(00)00687-2

[5] Glasser SP , Halberg DL , Sands C , Gamboa CM , Muntner P , Safford M . Is pulse pressure an independent risk factor for incident acute coronary heart disease events? The REGARDS study. Am J Hypertens. 2014;27:555–563. 24029164 10.1093/ajh/hpt168 PMC4014855

[6] Fernandes VR , Polak JF , Cheng S , Rosen BD , Carvalho B , Nasir K , McClelland R , Hundley G , Pearson G , O'Leary DH , Bluemke DA , Lima JA . Arterial stiffness is associated with regional ventricular systolic and diastolic dysfunction: the Multi‐Ethnic Study of Atherosclerosis. Arterioscler Thromb Vasc Biol. 2008;28:194–201. 17962621 10.1161/ATVBAHA.107.156950

[7] Laurent S , Cockcroft J , Van Bortel L , Boutouyrie P , Giannattasio C , Hayoz D , Pannier B , Vlachopoulos C , Wilkinson I , Struijker‐Boudier H ; European Network for Non‐invasive Investigation of Large Arteries . Expert consensus document on arterial stiffness: methodological issues and clinical applications. Eur Heart J. 2006;27:2588–2605. 17000623 10.1093/eurheartj/ehl254

[8] Vlachopoulos C , Aznaouridis K , Stefanadis C . Prediction of cardiovascular events and all‐cause mortality with arterial stiffness: a systematic review and meta‐analysis. J Am Coll Cardiol. 2010;55:1318–1327. 20338492 10.1016/j.jacc.2009.10.061

[9] Alty SR , Angarita‐Jaimes N , Millasseau SC , Chowienczyk PJ . Predicting arterial stiffness from the digital volume pulse waveform. IEEE Trans Biomed Eng. 2007;54:2268–2275. 18075043 10.1109/tbme.2007.897805

[10] Millasseau SC , Kelly RP , Ritter JM , Chowienczyk PJ . Determination of age‐related increases in large artery stiffness by digital pulse contour analysis. Clin Sci (Lond). 2002;103:371–377. 12241535 10.1042/cs1030371

[11] UK Biobank . UK Biobank: register & apply. Available at: http://www.ukbiobank.ac.uk/register-apply/. Accessed October 29, 2017.

[12] Sudlow C , Gallacher J , Allen N , Beral V , Burton P , Danesh J , Downey P , Elliott P , Green J , Landray M , Liu B , Matthews P , Ong G , Pell J , Silman A , Young A , Sprosen T , Peakman T , Collins R . UK Biobank: an open access resource for identifying the causes of a wide range of complex diseases of middle and old age. PLoS Med. 2015;12:e1001779. 25826379 10.1371/journal.pmed.1001779 PMC4380465

[13] UK Biobank . UK Biobank: protocol for a large‐scale prospective epidemiological resource. Available at: http://www.ukbiobank.ac.uk/wp-content/uploads/2011/11/UK-Biobank-Protocol.pdf. Accessed December 15, 2015.

[14] UK Biobank . UK Biobank ethics and governance framework. Available at: http://tinyurl.com/ukbioethicsandgovernance. Accessed December 15, 2015.

[15] UK Biobank . UK Biobank arterial pulse‐wave velocity. Available at: https://biobank.ctsu.ox.ac.uk/crystal/docs/Pulsewave.pdf. Accessed November 20, 2015.

[16] Sollinger D , Mohaupt MG , Wilhelm A , Uehlinger D , Frey FJ , Eisenberger U . Arterial stiffness assessed by digital volume pulse correlates with comorbidity in patients with ESRD. Am J Kidney Dis. 2006;48:456–463. 16931219 10.1053/j.ajkd.2006.05.014

[17] WHO . International Classification of Diseases (ICD). Available at: http://www.who.int/classifications/icd/en/. Accessed January 10, 2016.

[18] National Health Service . NHS Classifcations OPCS‐4. Available at: https://isd.digital.nhs.uk/trud3/user/guest/group/61/pack/10. Accessed November 30, 2017.

[19] UK Biobank . UK Biobank blood pressure. Available at: https://biobank.ctsu.ox.ac.uk/crystal/docs/Bloodpressure.pdf. Accessed December 28, 2015.

[20] Coleman A , Freeman P , Steel S , Shennan A . Validation of the Omron 705IT (HEM‐759‐E) oscillometric blood pressure monitoring device according to the British Hypertension Society protocol. Blood Press Monit. 2006;11:27–32. 16410738 10.1097/01.mbp.0000189788.05736.5f

[21] Stang A , Moebus S , Mohlenkamp S , Dragano N , Schmermund A , Beck EM , Siegrist J , Erbel R , Jockel KH ; Heinz Nixdorf Recall Study Investigative Group . Algorithms for converting random‐zero to automated oscillometric blood pressure values, and vice versa. Am J Epidemiol. 2006;164:85–94. 16675536 10.1093/aje/kwj160

[22] Pencina MJ , D'Agostino RB Sr , D'Agostino RB Jr , Vasan RS . Evaluating the added predictive ability of a new marker: from area under the ROC curve to reclassification and beyond. Stat Med. 2008;72:157–172; discussion 207‐12. 10.1002/sim.2929 17569110

[23] D'Agostino RB Sr , Vasan RS , Pencina MJ , Wolf PA , Cobain M , Massaro JM , Kannel WB . General cardiovascular risk profile for use in primary care: the Framingham Heart Study. Circulation. 2008;117:743–753. 18212285 10.1161/CIRCULATIONAHA.107.699579

[24] Franklin SS , Gustin W IV , Wong ND , Larson MG , Weber MA , Kannel WB , Levy D . Hemodynamic patterns of age‐related changes in blood pressure. The Framingham Heart Study. Circulation. 1997;96:308–315. 9236450 10.1161/01.cir.96.1.308

[25] Lantelme P , Mestre C , Lievre M , Gressard A , Milon H . Heart rate: an important confounder of pulse wave velocity assessment. Hypertension. 2002;39:1083–1087. 12052846 10.1161/01.hyp.0000019132.41066.95

[26] Greenland P , Daviglus ML , Dyer AR , Liu K , Huang CF , Goldberger JJ , Stamler J . Resting heart rate is a risk factor for cardiovascular and noncardiovascular mortality: the Chicago Heart Association Detection Project in Industry. Am J Epidemiol. 1999;149:853–862. 10221322 10.1093/oxfordjournals.aje.a009901

[27] Westerhof N , O'Rourke MF . Haemodynamic basis for the development of left ventricular failure in systolic hypertension and for its logical therapy. J Hypertens. 1995;13:943–952. 8586828 10.1097/00004872-199509000-00002

[28] Safar ME . Systolic blood pressure, pulse pressure and arterial stiffness as cardiovascular risk factors. Curr Opin Nephrol Hypertens. 2001;10:257–261. 11224702 10.1097/00041552-200103000-00015

[29] Mitchell GF , Hwang SJ , Vasan RS , Larson MG , Pencina MJ , Hamburg NM , Vita JA , Levy D , Benjamin EJ . Arterial stiffness and cardiovascular events: the Framingham Heart Study. Circulation. 2010;121:505–511. 20083680 10.1161/CIRCULATIONAHA.109.886655 PMC2836717

[30] Blacher J , Staessen JA , Girerd X , Gasowski J , Thijs L , Liu L , Wang JG , Fagard RH , Safar ME . Pulse pressure not mean pressure determines cardiovascular risk in older hypertensive patients. Arch Intern Med. 2000;160:1085–1089. 10789600 10.1001/archinte.160.8.1085

[31] Mattace‐Raso FU , van der Cammen TJ , Hofman A , van Popele NM , Bos ML , Schalekamp MA , Asmar R , Reneman RS , Hoeks AP , Breteler MM , Witteman JC . Arterial stiffness and risk of coronary heart disease and stroke: the Rotterdam Study. Circulation. 2006;113:657–663. 16461838 10.1161/CIRCULATIONAHA.105.555235

[32] Sutton‐Tyrrell K , Najjar SS , Boudreau RM , Venkitachalam L , Kupelian V , Simonsick EM , Havlik R , Lakatta EG , Spurgeon H , Kritchevsky S , Pahor M , Bauer D , Newman A ; Health ABC Study . Elevated aortic pulse wave velocity, a marker of arterial stiffness, predicts cardiovascular events in well‐functioning older adults. Circulation. 2005;111:3384–3390. 15967850 10.1161/CIRCULATIONAHA.104.483628

[33] Vakalis K , Bechlioulis A , Naka KK , Chatzikyriakidou A , Gartzonika K , Vezyraki P , Kolios G , Pappas K , Katsouras CS , Georgiou I , Michalis LK . Role of 9p21 and 2q36 variants and arterial stiffness in the prediction of coronary artery disease. Eur J Clin Invest. 2014;44:784–794. 24942486 10.1111/eci.12295

[34] Franklin SS , Khan SA , Wong ND , Larson MG , Levy D . Is pulse pressure useful in predicting risk for coronary heart Disease? The Framingham Heart Study. Circulation. 1999;100:354–360. 10421594 10.1161/01.cir.100.4.354

[35] Tsao CW , Lyass A , Larson MG , Levy D , Hamburg NM , Vita JA , Benjamin EJ , Mitchell GF , Vasan RS . Relation of central arterial stiffness to incident heart failure in the community. J Am Heart Assoc. 2015;4:e002189 DOI: 10.1161/JAHA.115.002189. 26597152 10.1161/JAHA.115.002189 PMC4845230

[36] Haider AW , Larson MG , Franklin SS , Levy D ; Framingham Heart Study . Systolic blood pressure, diastolic blood pressure, and pulse pressure as predictors of risk for congestive heart failure in the Framingham Heart Study. Ann Intern Med. 2003;138:10–16. 12513039 10.7326/0003-4819-138-1-200301070-00006

[37] Chae CU , Pfeffer MA , Glynn RJ , Mitchell GF , Taylor JO , Hennekens CH . Increased pulse pressure and risk of heart failure in the elderly. JAMA. 1999;281:634–639. 10029125 10.1001/jama.281.7.634

[38] Domanski MJ , Davis BR , Pfeffer MA , Kastantin M , Mitchell GF . Isolated systolic hypertension: prognostic information provided by pulse pressure. Hypertension. 1999;34:375–380. 10489379 10.1161/01.hyp.34.3.375

[39] Zheng L , Sun Z , Li J , Zhang R , Zhang X , Liu S , Li J , Xu C , Hu D , Sun Y . Pulse pressure and mean arterial pressure in relation to ischemic stroke among patients with uncontrolled hypertension in rural areas of China. Stroke. 2008;39:1932–1937. 18451345 10.1161/STROKEAHA.107.510677

[40] Glynn RJ , Chae CU , Guralnik JM , Taylor JO , Hennekens CH . Pulse pressure and mortality in older people. Arch Intern Med. 2000;160:2765–2772. 11025786 10.1001/archinte.160.18.2765

[41] Lee ML , Rosner BA , Weiss ST . Relationship of blood pressure to cardiovascular death: the effects of pulse pressure in the elderly. Ann Epidemiol. 1999;9:101–107. 10037553 10.1016/s1047-2797(98)00034-9

[42] Gupta RM , Hadaya J , Trehan A , Zekavat SM , Roselli C , Klarin D , Emdin CA , Hilvering CRE , Bianchi V , Mueller C , Khera AV , Ryan RJH , Engreitz JM , Issner R , Shoresh N , Epstein CB , de Laat W , Brown JD , Schnabel RB , Bernstein BE , Kathiresan S . A genetic variant associated with five vascular diseases is a distal regulator of endothelin‐1 gene expression. Cell. 2017;170:522–533.e15. 28753427 10.1016/j.cell.2017.06.049 PMC5785707

