# Supplementary material for: Relationship of Arterial Stiffness Index and Pulse Pressure With Cardiovascular Disease and Mortality
Source: J Am Heart Assoc. 2018 Jan 22;7(2):e007621. doi: 10.1161/JAHA.117.007621 (PMC5850166; doi:10.1161/JAHA.117.007621)
Supplement: Supplementary file 1 — Table S1. Variable Definitions Used in UK Biobank Table S2. Baseline Characteristics of Included and Excluded UK Biobank Participants Table S3. Independent Association of ASI and PP With Disease Table S4. Harrell's C‐Indices and Postestimation Analyses of Models for Risk Prediction of Disease and Mortality With and Without ASI or PP Table S5. Association of ASI With Non‐CVD/Noncancer and Non‐CVD Cancer Mortality Table S6. Independent Association of ASI and PP With Mortality Table S7. Association of ASI and PP With Diseases and Mortality, Additionally Adjusted for Heart Rate Table S8. Harrell's C‐Indices for ASI and PP in Diabetics and Nondiabetics Table S9. Harrell's C‐Indices for ASI and PP in Participants With and Without Hypertension Table S10. Harrell's C‐Indices for ASI and PP in Participants Younger or Older Than the Median 56.8 Years Table S11. Harrell's C‐Indices for ASI and PP in Participants With Lower or Higher FRS Than the Median Table S12. Harrell's C‐Indices for ASI and PP in Men and Women Separately Figure S1. Shown are the inclusion and exclusion criteria for the sample selection strategy used in our analyses. ASI indicates arterial stiffness index; PP, pulse pressure. Figure S2. Shown are regression spline models in which the red line with gray‐colored 95% confidence intervals (CI) represents the linear regression of ASI and pulse pressure over increasing age (per 5 years) for men and women separately. ASI indicates arterial stiffness index. Figure S3. Shown are cumulative new‐onset myocardial infarction, coronary heart disease, heart failure, and stroke in (%) divided by the median ASI. All curves were adjusted for age and sex. Log‐Rank testing shows significant differences for both myocardial infarction and coronary heart disease. ASI indicates arterial stiffness index. Figure S4. Shown are cumulative new‐onset myocardial infarction, coronary heart disease, and stroke in (%) divided by the median PP. All curves were adjusted for age and sex. Log‐R [file JAH3-7-e007621-s001.pdf]

SUPPLEMENTAL MATERIAL

Table S1. Variable definitions used in UK Biobank

Variable	ICD-9	ICD-10	OPCS-4	Self-reported fields
Cardiovascular disease	3361, 36231, 36232, 39-44	I00-I78, G951, H341, H342, O10, S066, Z951, Z955	A052-A054, K01, K02, K04, K07, K09-K14, K18-K31, K33-K35, K37, K38, K40-K50, K52-K55, K571-K576, K621-K623, K64, K68, K75, K77, L16, L18-L23, L25, L263, L265, L266, L268-L301, L303, L304, L311, L313, L314, L33-L351, L353, L355, L37-L381, L383, L384, L391, L392, L395, L41, L421, L424, L428-L432, L435, L45, L461, L464, L471, L474, L48-L542, L544, L56-L632, L635, L638, L639, L651-L653, L661, L662, L665, L667, L68, L701, L705, L711, L712, L715-L717, O01-O03, X503, X504, X508, X509	3267, 4056, 5529(1), 5540(1), 6150(2,3), 20002(1067, 1074-1079, 1081, 1082, 1086, 1087, 1425, 1426, 1471, 1479, 1483-1492, 1583-1591), 20004(1069, 1070, 1095, 1097-1103, 1108, 1523, 1553)
Coronary heart disease	410, 412, 414	I21-25, Z951, Z955	K40-K46, K49, K50, K75	6150(1), 3894, 20004(1070, 1095, 1523)
Myocardial infarction	410, 412	I21-I23, I252		20002(1075)
Heart failure	428	I110, I130, I132, I50		20002(1076)
Hypertension	401-405	I10-I13, I15, O10		2966, 6150(4), 6153(2), 6177(2), 20002(1065, 1072)
Stroke	3361, 36231, 36232, 430, 431, 4329, 43301, 43311, 43321, 43331, 43381, 43391, 434, 436	I60, I61, I629, I63, I64, I678, I690, I693, G951, H341, H342, S066	A052-A054, L351, L353, L343	6150(3), 4056, 20002(1081, 1491, 1583, 1086)
Diabetes mellitus	250	E10-E14		2976, 6153(3), 6177(3), 20002(1220, 1222, 1223)
Hyperlipidemia	272	E78		6153(1), 6177(1), 1473
Cancer	21-23, 141, 142, 144, 146, 150-157, 159-162, 164, 170-174, 179, 180, 182-191, 193, 195, 196, 198-202, 204, 205, V10	C00-C96, D00-D48, Z85		20001

Variable definitions constructed using ICD-9, ICD-10 and OPCS-4 codes as well as self-reported data fields with disease- or procedure-specific codes between brackets are shown.
Abbreviations: ICD, International Classification of Diseases; OPCS, Office of Population, Censuses and Surveys: Classification of interventions and Procedures

Table S2. Baseline characteristics of included and excluded UK Biobank participants

Characteristics	Included participants n=169,613	Excluded participants n=333,042
	**Mean±SD or n (%)**	**Mean±SD or n (%)**
Males	77,708 (45.8)	151,470 (45.5)
Age, y	56.77±8.16	56.41±8.06
Heart rate, bpm	68.72±11.01	69.84±11.31
Body mass index, kg/m^2	27.46±4.82	27.43±4.80
Systolic blood pressure, mm Hg	132.92±17.78	133.47±18.07
Diastolic blood pressure, mm Hg	81.94±8.37	82.13±8.68
Mean arterial pressure, mm Hg	98.94±10.65	99.24±10.93
Hypertension	52,885 (31.2)	102,330 (30.7)
Diabetes	10,267 (6.1)	17,591 (5.3)
Hyperlipidemia	34,723 (20.5)	61,306 (18.4)
Past or current smoker	100,590 (59.3)	198,302 (59.5)
Ethnicity		
White	153,931 (90.8)	318,898 (95.8)
Asian	6,586 (3.9)	4,870 (1.5)
Black	4,508 (2.7)	3,558 (1.1)
Mixed	1,294 (0.8)	1.664 (0.5)
Other/Unknown	3,294 (1.9)	4,052 (1.2)

Means with Standard Deviation (SD) or counts with percentages are given per characteristic.

Table S3. The independent association of ASI and PP with disease

	Cardiovascular Disease n_{total}=141,571 n_{event}=18,190 (12.8%)			Myocardial Infarction n_{total}=165,589 n_{event}=1,587 (1.0%)			Coronary Heart Disease n_{total}=162,543 n_{event}=4,326 (2.7%)	
	**HR (95%CI)**	***P value***		**HR (95%CI)**	***P value***		**HR (95%CI)**	***P value***
**ASI**								
Model 3	1.04 (1.03 to 1.06)	<0.001	Model 3	1.13 (1.07 to 1.18)	<0.001	Model 3	1.08 (1.05 to 1.11)	<0.001
Model 3*	1.05 (1.03 to 1.06)	<0.001	Model 3*	1.14 (1.09 to 1.20)	<0.001	Model 3*	1.09 (1.06 to 1.12)	<0.001
**PP**								
Model 3	1.05 (1.03 to 1.07)	<0.001	Model 3	1.11 (1.04 to 1.19)	0.001	Model 3	1.14 (1.09 to 1.18)	<0.001
Model 3†	1.06 (1.04 to 1.08)	<0.001	Model 3†	1.14 (1.06 to 1.21)	<0.001	Model 3†	1.15 (1.11 to 1.20)	<0.001

Table S3. Continued

	Heart Failure n_{total}=168,751 n_{event}=1,192 (0.7%)			Stroke n_{total}=166,954 n_{event}=1,319 (0.8%)	
	**HR (95%CI)**	***P value***		**HR (95%CI)**	***P value***
**ASI**					
Model 3	1.07 (1.01 to 1.13)	0.02	Model 2	1.08 (1.02 to 1.14)	<0.01
Model 3*	1.09 (1.03 to 1.15)	<0.01	Model 2*	1.08 (1.03 to 1.14)	<0.01
**PP**					
Model 3	1.21 (1.13 to 1.30)	<0.001	Model 2	1.16 (1.10 to 1.22)	<0.001
Model 3†	1.23 (1.14 to 1.32)	<0.001	Model 2†	1.16 (1.10 to 1.22)	<0.001

Hazard ratios (HR) with 95% Confidence Interval (CI) estimated using ASI per SD change in m/s and PP per SD change in mm Hg are shown for cardiovascular disease, myocardial infarction, coronary heart disease, heart failure and stroke. Shown are the outcomes for the largest significant model and additional adjustment for PP or ASI. Model 2: Adjusted for age and sex. Model 3: Model 2 + mean arterial pressure, diabetes, smoking and BMI.
Abbreviations: ASI, Arterial Stiffness Index; NA, Not Applicable; PP, Pulse Pressure
* Additionally adjusted for PP
† Additionally adjusted for ASI

Table S4. Harrell's C-indices and post estimation analyses of models for risk prediction of disease and mortality with and without ASI or PP

	CVD n_{total}=141,571 n_{event}=18,190 (12.8%)		Myocardial Infarction n_{total}=165,589 n_{event}=1,587 (1.0%)		Coronary Heart Disease n_{total}=162,543 n_{event}=4,326 (2.7%)		Heart Failure n_{total}=168,751 n_{event}=1,192 (0.7%)	
	**Harrell's C-index**	***P value***	**Harrell's C-index**	***P value***	**Harrell's C-index**	***P value***	**Harrell's C-index**	***P value***
**ASI**								
Model 3+ASI	0.723	<0.05	0.738	0.01	0.725	0.01	0.772	0.34
Model 3–ASI	0.723		0.736		0.724		0.772	
Model 3+PP+ASI	0.723	0.02	0.738	0.02	0.726	<0.01	0.773	0.33
Model 3+PP-ASI	0.723		0.736		0.725		0.773	
**PP**								
Model 3+PP	0.723	<0.001	0.736	0.94	0.725	0.03	0.773	0.40
Model 3–PP	0.722		0.736		0.724		0.772	
Model 3+ASI+PP	0.723	<0.001	0.738	0.93	0.726	0.02	0.773	0.37
Model 3+ASI–PP	0.723		0.738		0.725		0.772	

Table S4. Continued.

	Stroke n_{total}=166,954 n_{event}=1,319 (0.8%)		All-cause Mortality n_{total}=169,613 n_{event}=3.678 (2.2%)		CVD Mortality n_{total}=169,613 n_{event}=1,180 (0.7%)		Non-CVD Mortality n_{total}=169,613 n_{event}=2,498 (1.5%)	
	**Harrell's C-index**	***P value***	**Harrell's C-index**	***P value***	**Harrell's C-index**	***P value***	**Harrell's C-index**	***P value***
**ASI**								
Model 3+ASI	NA	NA	0.715*	0.01*	0.795*	0.10*	0.680*	0.07*
Model 3–ASI	NA		0.714*		0.794*		0.679*	
Model 3+PP+ASI	NA	NA	0.715*	0.01*	0.795*	0.12*	0.680*	0.06*
Model 3+PP-ASI	NA		0.714*		0.793*		0.679*	
**PP**								
Model 3+PP	NA	NA	NA	NA	0.793*	0.79*	NA	NA
Model 3–PP	NA		NA		0.794*		NA	
Model 3+ASI+PP	NA	NA	NA	NA	0.795*	0.82*	NA	NA
Model 3+ASI–PP	NA		NA		0.795*		NA	

Harrell's C-indices are given for models with (+) and without (-) ASI or PP and for models with ASI or PP with or without additional adjustment for PP or ASI respectively. Post estimation analysis *P* values for the difference between the predictive values of the models are given. Model 3: adjusted for age, sex, mean arterial pressure, diabetes, smoking and body mass index. Model 4: Model 3 + history of CVD, myocardial infarction, coronary heart disease, heart failure and stroke.
Abbreviations: ASI, Arterial Stiffness Index; CVD, Cardiovascular Disease; PP, Pulse Pressure. * Results are given for Model 4 instead of Model 3.

Table S5. Association of ASI with non-CVD/non-cancer and non-CVD cancer mortality

	Non-CVD/Non-Cancer mortality n_{total}=169,613 n_{event}=465 (0.3%)		Non-CVD Cancer mortality n_{total}=169,613 n_{event}=2,033 (1.2%)	
	**Hazard Ratio (95%CI)**	***P* value**	**Hazard Ratio (95%CI)**	***P* value**
**ASI**				
Model 1	1.25 (1.15 to 1.36)	<0.001	1.22 (1.17 to 1.27)	<0.001
Model 2	1.10 (1.01 to 1.20)	0.04	1.07 (1.02 to 1.12)	<0.01
Model 3	1.12 (1.02 to 1.22)	0.02	1.06 (1.02 to 1.11)	<0.01
Model 4	1.12 (1.02 to 1.22)	0.02	1.06 (1.02 to 1.11)	<0.01

Hazard ratios with 95% Confidence Interval (CI) estimated using ASI per SD change in m/s are shown per model for non-CVD/non-cancer and non-CVD cancer mortality. Model 1: Univariate (unadjusted). Model 2: Adjusted for age and sex. Model 3: Model 2 + mean arterial pressure, diabetes, smoking and body mass index, Model 4: Model 3 + history of CVD, myocardial infarction, coronary heart disease, heart failure, and stroke.
Abbreviations: ASI, Arterial Stiffness Index; CVD, Cardiovascular Disease.

Table S6. Independent association of ASI and PP with mortality

	All-cause Mortality<br>n_{total}=169,613<br>n_{event}=3.678 (2.2%)			CVD Mortality<br>n_{total}=169,613<br>n_{event}=1,180 (0.7%)			Non-CVD Mortality<br>n_{total}=169,613<br>n_{event}=2,498 (1.5%)	
	**HR (95%CI)**	***P* value**		**HR (95%CI)**	***P* value**		**HR (95%CI)**	***P* value**
**ASI**								
Model 4	1.08 (1.05 to 1.12)	<0.001	Model 4	1.11 (1.05 to 1.17)	<0.001	Model 4	1.07 (1.03 to 1.11)	0.001
Model 4*	1.08 (1.05 to 1.12)	<0.001	Model 4*	1.12 (1.02 to 1.18)	<0.001	Model 4*	1.07 (1.03 to 1.11)	<0.001
**PP**								
Model 3	1.05 (1.00 to 1.09)	0.04	Model 4	1.10 (1.02 to 1.18)	0.02	Model 1	1.25 (1.21 to 1.30)	<0.001
Model 3†	1.06 (1.01 to 1.11)	0.01	Model 4†	1.11 (1.04 to 1.20)	<0.01	Model 1†	1.23 (1.19 to 1.28)	<0.001

Hazard ratios (HR) with 95% Confidence Interval (CI) estimated using ASI per SD change in m/s and PP per SD change in mm Hg are shown for all-cause, CVD and non-CVD mortality. Shown are the outcomes for the largest significant model and additional adjustment for PP or ASI. Model 1: Univariate (unadjusted). Model 3: Adjusted for age, sex, mean arterial pressure, smoking and body mass index. Model 4: Model 3 + history of CVD, myocardial infarction, coronary heart disease, heart failure and stroke.

Abbreviations: ASI, Arterial Stiffness Index; CVD, Cardiovascular Disease; PP, Pulse Pressure

* Additionally adjusted for PP

† Additionally adjusted for ASI

Table S7. Association of ASI and PP with diseases and mortality, additionally adjusted for heart rate

	Cardiovascular Disease<br>n_{total}=141,571<br>n_{event}=18,190 (12.8%)			Myocardial Infarction<br>n_{total}=165,589<br>n_{event}=1,587 (1.0%)			Coronary Heart Disease<br>n_{total}=162,543<br>n_{event}=4,326 (2.7%)	
	**HR (95%CI)**	***P value***		**HR (95%CI)**	***P value***		**HR (95%CI)**	***P value***
**ASI**								
Model 3	1.04 (1.03 to 1.06)	<0.001	Model 3	1.13 (1.07 to 1.18)	<0.001	Model 3	1.08 (1.05 to 1.11)	<0.001
Model 3*	1.04 (1.03 to 1.06)	<0.001	Model 3*	1.13 (1.07 to 1.18)	<0.001	Model 3*	1.08 (1.05 to 1.11)	<0.001
**PP**								
Model 3	1.05 (1.03 to 1.07)	<0.001	Model 3	1.11 (1.04 to 1.19)	0.001	Model 3	1.14 (1.09 to 1.18)	<0.001
Model 3*	1.06 (1.04 to 1.08)	<0.001	Model 3*	1.14 (1.07 to 1.22)	<0.001	Model 3*	1.15 (1.11 to 1.20)	<0.001

Table S7. Continued.

	**Heart Failure**<br>n_{total}=168,751<br>n_{event}=1,192 (0.7%)			**Stroke**<br>n_{total}=166,954<br>n_{event}=1,319 (0.8%)			**All-cause Mortality**<br>n_{total}=169,613<br>n_{event}=3.678 (2.2%)	
	**HR (95%CI)**	***P value***		**HR (95%CI)**	***P value***		**HR (95%CI)**	***P value***
**ASI**								
Model 3	1.07 (1.01 to 1.13)	0.02	Model 2	1.08 (1.02 to 1.14)	<0.01	Model 4	1.08 (1.05 to 1.12)	<0.001
Model 3*	1.06 (1.00 to 1.12)	0.04	Model 2*	1.07 (1.01 to 1.13)	0.01	Model 4*	1.06 (1.02 to 1.09)	<0.001
**PP**								
Model 3	1.21 (1.13 to 1.30)	<0.001	Model 2	1.16 (1.10 to 1.22)	<0.001	Model 3	1.05 (1.00 to 1.09)	0.04
Model 3*	1.27 (1.18 to 1.37)	<0.001	Model 2*	1.16 (1.10 to 1.23)	<0.001	Model 3*	1.16 (1.11 to 1.21)	<0.001

Table S7. Continued.

	CVD Mortality<br>n_{total}=169,613<br>n_{event}=1,180 (0.7%)			Non-CVD Mortality<br>n_{total}=169,613<br>n_{event}=2,498 (1.5%)	
	**HR (95%CI)**	***P value***		**HR (95%CI)**	***P value***
**ASI**					
Model 4	1.11 (1.05 to 1.17)	<0.001	Model 4	1.07 (1.03 to 1.11)	<0.001
Model 4*	1.08 (1.02 to 1.14)	<0.01	Model 4*	1.05 (1.01 to 1.09)	0.02
**PP**					
Model 4	1.10 (1.02 to 1.18)	0.02	Model 1	1.25 (1.21 to 1.30)	<0.001
Model 4*	1.24 (1.15 to 1.34)	<0.001	Model 1*	1.27 (1.22 to 1.31)	<0.001

Hazard ratios (HR) with 95% Confidence Interval (CI) estimated using ASI per SD change in m/s and PP per SD change in mm Hg are shown for disease and mortality outcomes. Shown are the outcomes for the largest significant model and additional adjustment for heart rate. Model 1: Univariate (unadjusted). Model 3: adjusted for age, sex, mean arterial pressure, diabetes, smoking and BMI. Model 4: Model 3 + history of cardiovascular disease, myocardial infarction, coronary heart disease, heart failure and stroke.
Abbreviations: ASI, Arterial Stiffness Index; PP, Pulse Pressure
* Additionally adjusted for heart rate

Table S8. Harrell's C-indices for ASI and PP in diabetics and non-diabetics

				Harrell's C index		
		N_{total}	N_{event}	**ASI**	**PP**	***P* value**
**No Diabetes**	Cardiovascular Disease	135,502	16,298	0.718	0.718	0.86
	Myocardial Infarction	156,206	1,351	0.733	0.730	0.02
	Coronary Heart Disease	153,803	3,722	0.718	0.717	0.56
	Heart Failure	158,704	929	0.753	0.753	0.84
	Stroke	157,124	1,129	0.706	0.706	0.77
	All-cause mortality	159,346	3,152	0.698	0.697	<0.05
	CVD mortality	159,346	921	0.759	0.757	0.21
	Non-CVD mortality	159,346	2,231	0.676	0.674	0.10
**Diabetes**	Cardiovascular Disease	6,069	1,892	0.620	0.623	0.02
	Myocardial Infarction	9,383	236	0.650	0.671	0.12
	Coronary Heart Disease	8,740	604	0.626	0.635	0.09
	Heart Failure	10,047	263	0.705	0.711	0.37
	Stroke	9,830	190	0.643	0.641	0.67
	All-cause mortality	10,267	526	0.657	0.661	0.25
	CVD mortality	10,267	259	0.687	0.702	0.09
	Non-CVD mortality	10,267	267	0.631	0.631	0.93

Harrell's C-indices for the largest model (Model 3 for disease and Model 4 for mortality outcomes) per subgroup. Abbreviations: ASI, Arterial Stiffness Index; PP, Pulse Pressure

Table S9. Harrell's C-indices for ASI and PP in participants with and without hypertension

				Harrell's C index		
		N_{total}	N_{event}	**ASI**	**PP**	***P* value**
**No Hypertension**	Cardiovascular Disease	110,417	7,754	0.712	0.711	0.39
	Myocardial Infarction	116,307	754	0.742	0.740	0.10
	Coronary Heart Disease	115,878	1,982	0.732	0.731	0.27
	Heart Failure	116,678	400	0.763	0.762	0.81
	Stroke	115,887	621	0.708	0.708	0.75
	All-cause mortality	116,728	1,870	0.694	0.693	0.11
	CVD mortality	116,728	428	0.756	0.755	0.56
	Non-CVD mortality	116,728	1,442	0.679	0.677	0.12
**Hypertension**	Cardiovascular Disease	31,154	10,436	0.600	0.600	0.52
	Myocardial Infarction	49,282	833	0.679	0.677	0.40
	Coronary Heart Disease	46,665	2,344	0.649	0.650	0.72
	Heart Failure	52,073	792	0.705	0.706	0.62
	Stroke	51,067	698	0.654	0.654	0.70
	All-cause mortality	52,885	1,808	0.680	0.680	0.80
	CVD mortality	52,885	752	0.729	0.730	0.74
	Non-CVD mortality	52,885	1,056	0.648	0.647	0.34

Harrell's C-indices for the largest model (Model 3 for disease and Model 4 for mortality outcomes) per subgroup. Abbreviations: ASI, Arterial Stiffness Index; PP, Pulse Pressure

Table S10. Harrell's C-indices for ASI and PP in participants younger or older than the median 56.8 years

				Harrell's C index		
		**N_{total}**	**N_{event}**	**ASI**	**PP**	***P* value**
**Young (≤56.8 y)**	Cardiovascular Disease	67,821	4,843	0.735	0.735	0.02
	Myocardial Infarction	73,782	367	0.743	0.736	0.06
	Coronary Heart Disease	73,355	947	0.733	0.731	0.21
	Heart Failure	74,306	189	0.739	0.735	0.59
	Stroke	73,737	248	0.647	0.645	0.54
	All-cause mortality	74,468	737	0.656	0.648	0.03
	CVD mortality	74,468	192	0.729	0.717	0.16
	Non-CVD mortality	74,468	545	0.636	0.630	0.15
**Old (>56.8 y)**	Cardiovascular Disease	73,750	13,347	0.660	0.662	<0.01
	Myocardial Infarction	91,807	1,220	0.694	0.692	0.40
	Coronary Heart Disease	89,188	3,379	0.662	0.663	0.19
	Heart Failure	94,445	1,003	0.716	0.716	0.85
	Stroke	93,217	1,071	0.653	0.653	0.96
	All-cause mortality	95,145	2,941	0.653	0.652	0.33
	CVD mortality	95,145	988	0.730	0.729	0.32
	Non-CVD mortality	95,145	1,953	0.617	0.616	0.34

Harrell's C-indices for the largest model (Model 3 for disease and Model 4 for mortality outcomes) per subgroup. Abbreviations: ASI, Arterial Stiffness Index; PP, Pulse Pressure

Table S11. Harrell's C-indices for ASI and PP in participants with lower or higher FRS than the median

				Harrell's C index		
		N_{total}	N_{event}	ASI	PP	*P* value
**FRS ≤9.29**	Cardiovascular Disease	78,122	5,536	0.706	0.706	0.12
	Myocardial Infarction	85,473	303	0.669	0.666	0.50
	Coronary Heart Disease	85,039	971	0.685	0.684	0.47
	Heart Failure	85,849	213	0.720	0.723	0.48
	Stroke	85,240	336	0.659	0.657	0.39
	All-cause mortality	85,974	963	0.654	0.650	0.10
	CVD mortality	85,974	189	0.684	0.678	0.18
	Non-CVD mortality	85,974	774	0.650	0.648	0.31
**FRS >9.29**	Cardiovascular Disease	63,449	12,654	0.648	0.649	0.14
	Myocardial Infarction	80,116	1,284	0.646	0.642	0.15
	Coronary Heart Disease	77,504	3,355	0.634	0.634	0.83
	Heart Failure	82,902	979	0.699	0.699	0.95
	Stroke	81,714	983	0.655	0.655	0.85
	All-cause mortality	83,639	2,715	0.658	0.657	0.54
	CVD mortality	83,639	991	0.705	0.703	0.28
	Non-CVD mortality	83,639	1,724	0.635	0.635	0.77

Harrell's C-indices for the largest model (Model 3 for disease and Model 4 for mortality outcomes) per subgroup. The FRS is calculated for a max of 5.92 years. Abbreviations: ASI, Arterial Stiffness Index; FRS, Framingham Risk; PP, Pulse Pressure

Table S12. Harrell's C-indices for ASI and PP in men and women separately

		N_{total}	N_{event}	Harrell's C index ASI	PP	*P* value
**Women**	Cardiovascular Disease	79,310	8,710	0.723	0.724	0.001
	Myocardial Infarction	91,040	439	0.704	0.701	0.38
	Coronary Heart Disease	90,117	1,500	0.711	0.713	0.11
	Heart Failure	91,704	404	0.753	0.760	0.04
	Stroke	90,785	545	0.702	0.703	0.41
	All-cause mortality	91,905	1,431	0.679	0.677	<0.05
	CVD mortality	91,905	327	0.734	0.733	0.50
	Non-CVD mortality	91,905	1,104	0.664	0.662	0.08
**Men**	Cardiovascular Disease	62,261	9,480	0.713	0.713	0.04
	Myocardial Infarction	74,549	1,148	0.670	0.667	0.22
	Coronary Heart Disease	72,426	2,826	0.680	0.680	0.63
	Heart Failure	77,047	788	0.748	0.745	0.15
	Stroke	76,169	774	0.702	0.702	0.49
	All-cause mortality	77,708	2,247	0.700	0.699	0.32
	CVD mortality	77,708	853	0.742	0.741	0.57
	Non-CVD mortality	77,708	1,394	0.675	0.674	0.36

Harrell's C-indices for the largest model (Model 3 for disease and Model 4 for mortality outcomes) per subgroup. Abbreviations: ASI, Arterial Stiffness Index; PP, Pulse Pressure

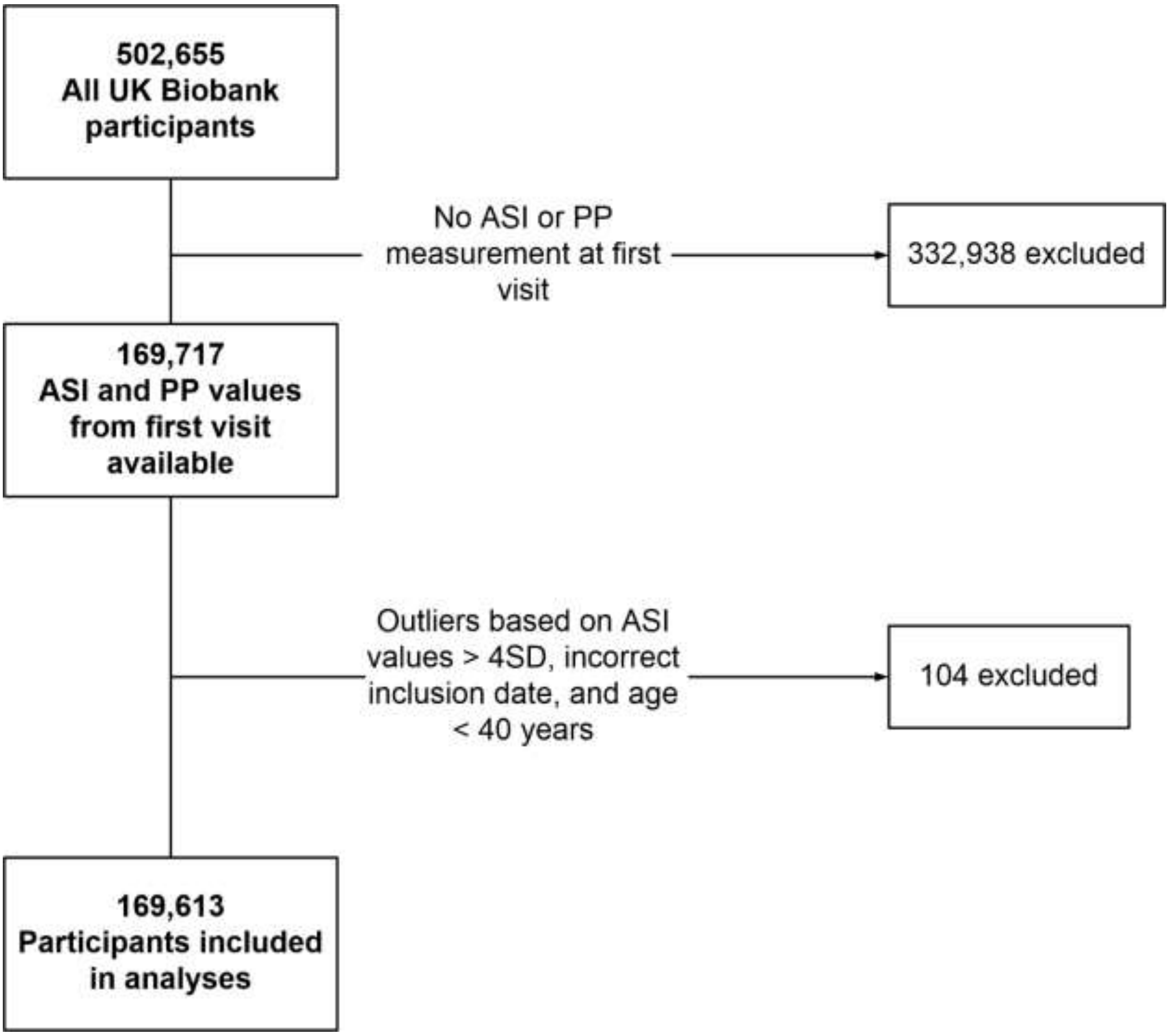

Figure S1. Shown are the in- and exclusion criteria for the sample selection strategy used in our analyses. Abbreviations: ASI, Arterial Stiffness Index; PP, Pulse Pressure; SD, Standard Deviation

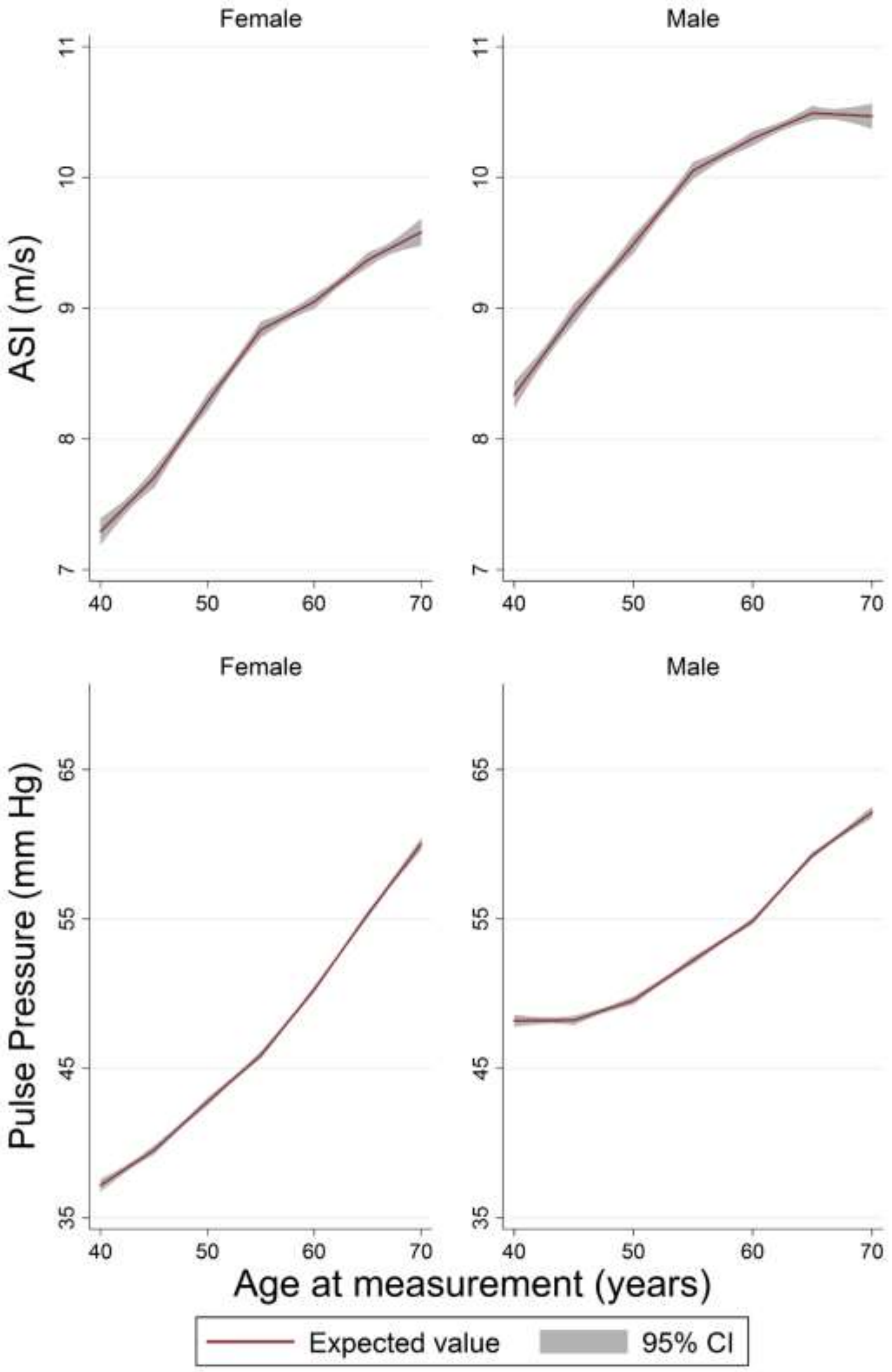

Figure S2. Shown are regression spline models in which the red line with gray colored 95% Confidence Intervals (CI) represents the linear regression of ASI and Pulse Pressure over increasing age (per 5 years) for men and women separately. Abbreviation: ASI, Arterial Stiffness Index

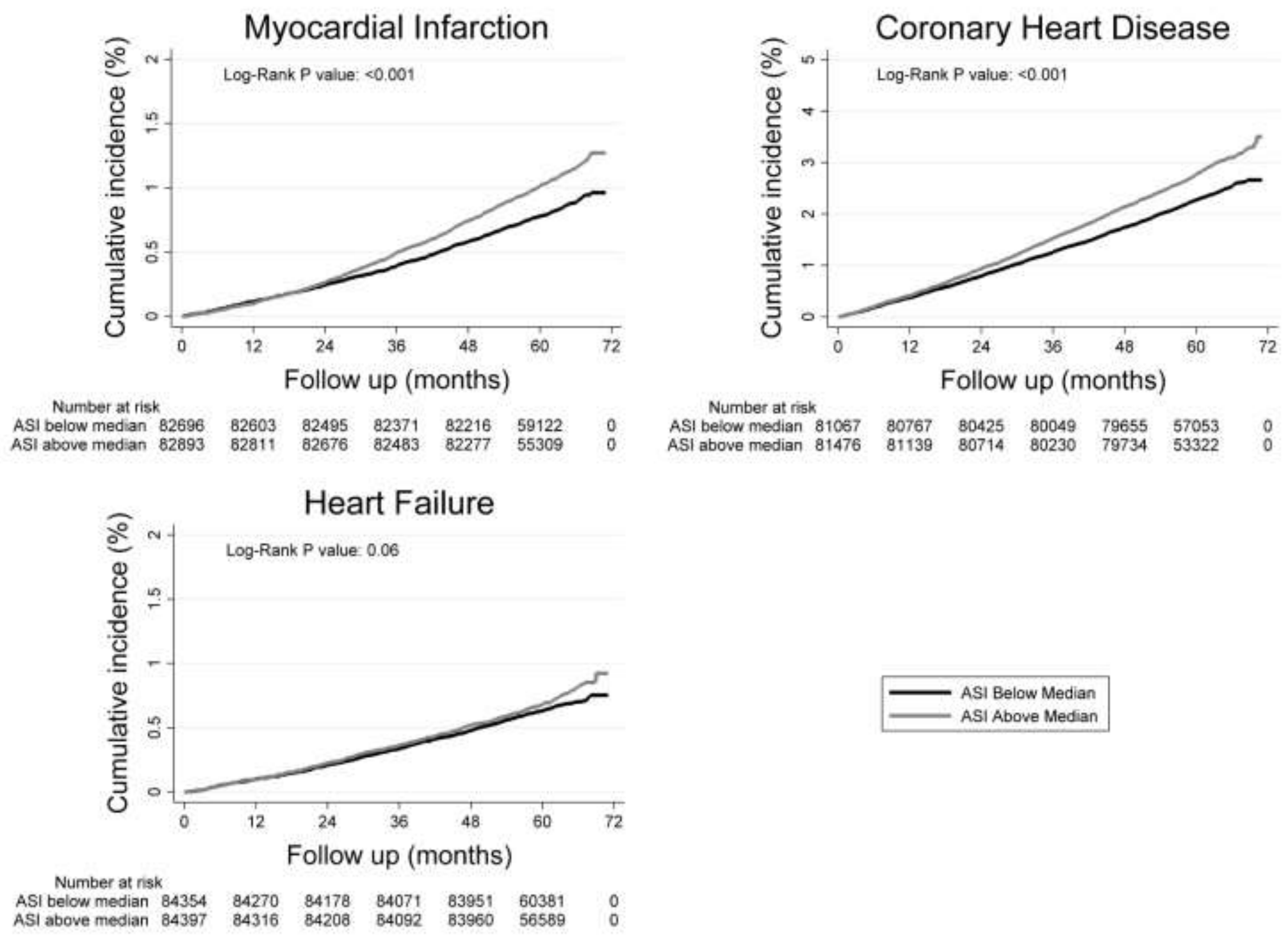

Figure S3. Shown are cumulative new-onset myocardial infarction, coronary heart disease, heart failure and stroke in (%) divided by the median ASI. All curves were adjusted for age and sex. Log-Rank testing shows significant differences for both myocardial infarction and coronary heart disease. Abbreviations: ASI, Arterial Stiffness index

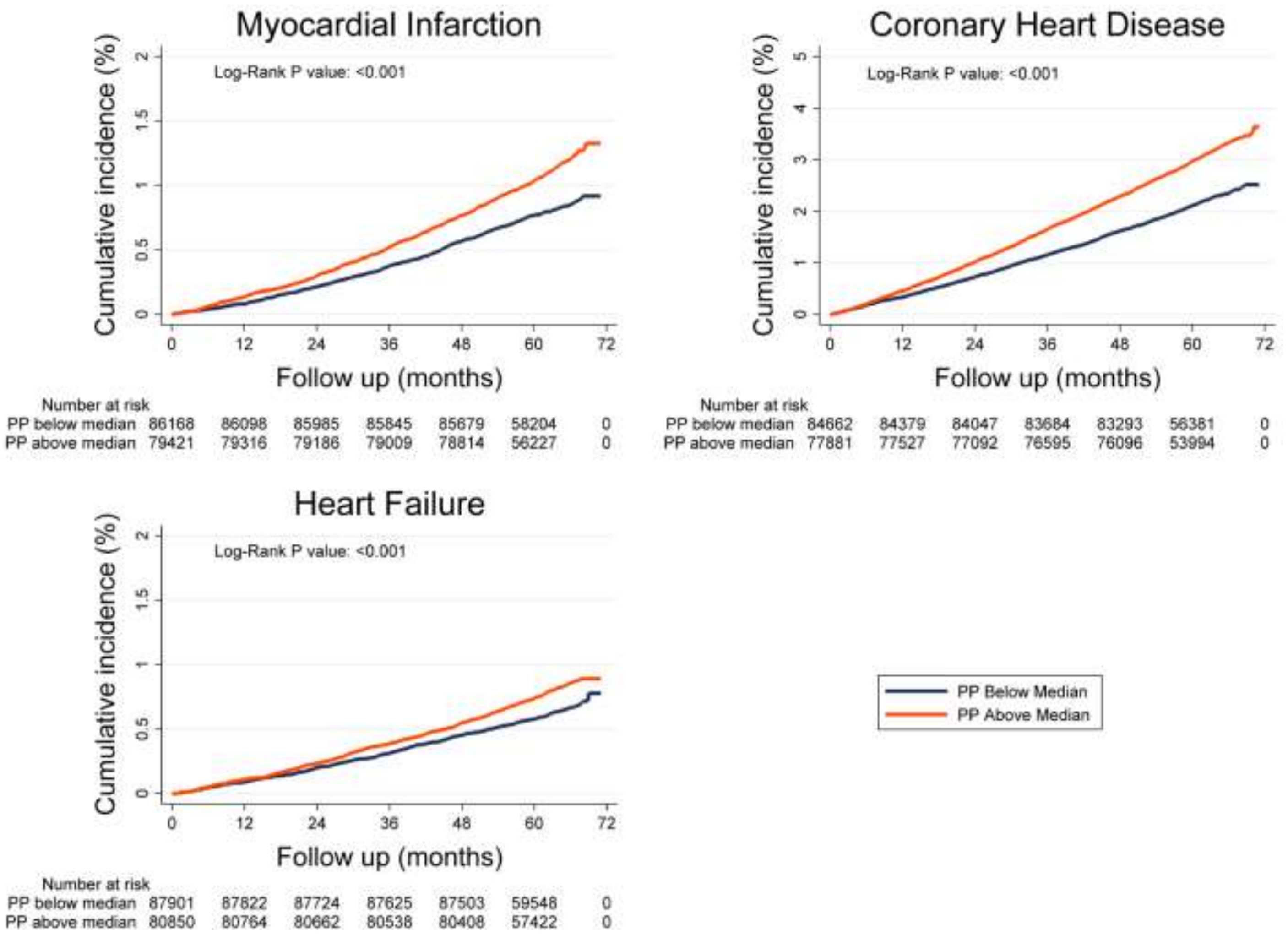

Figure S4. Shown are cumulative new-onset myocardial infarction, coronary heart disease and stroke in (%) divided by the median PP. All curves were adjusted for age and sex. Log-Rank testing shows significant differences for all. Abbreviations: PP, Pulse Pressure

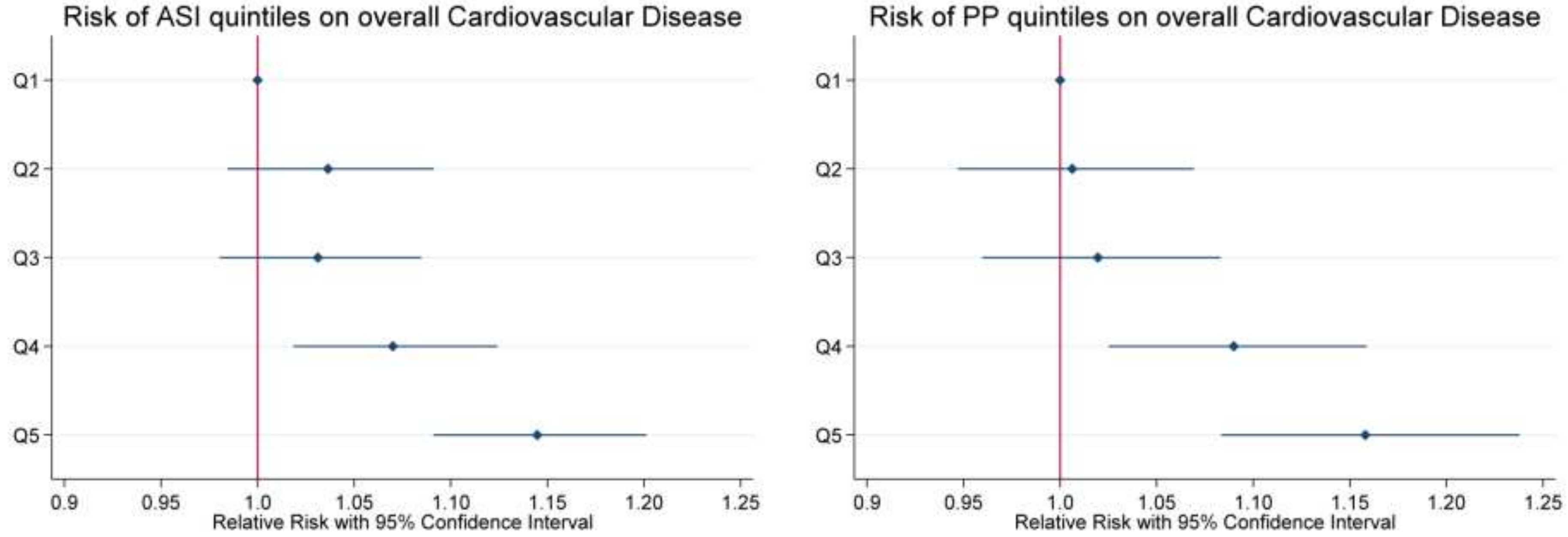

Figure S5. Shown are risk of overall cardiovascular disease for quintiles of arterial stiffness index and pulse pressure separately. The analyses were adjusted for age, sex, mean arterial pressure, diabetes, body mass index and smoking. Abbreviations: ASI, Arterial Stiffness Index; PP, Pulse Pressure

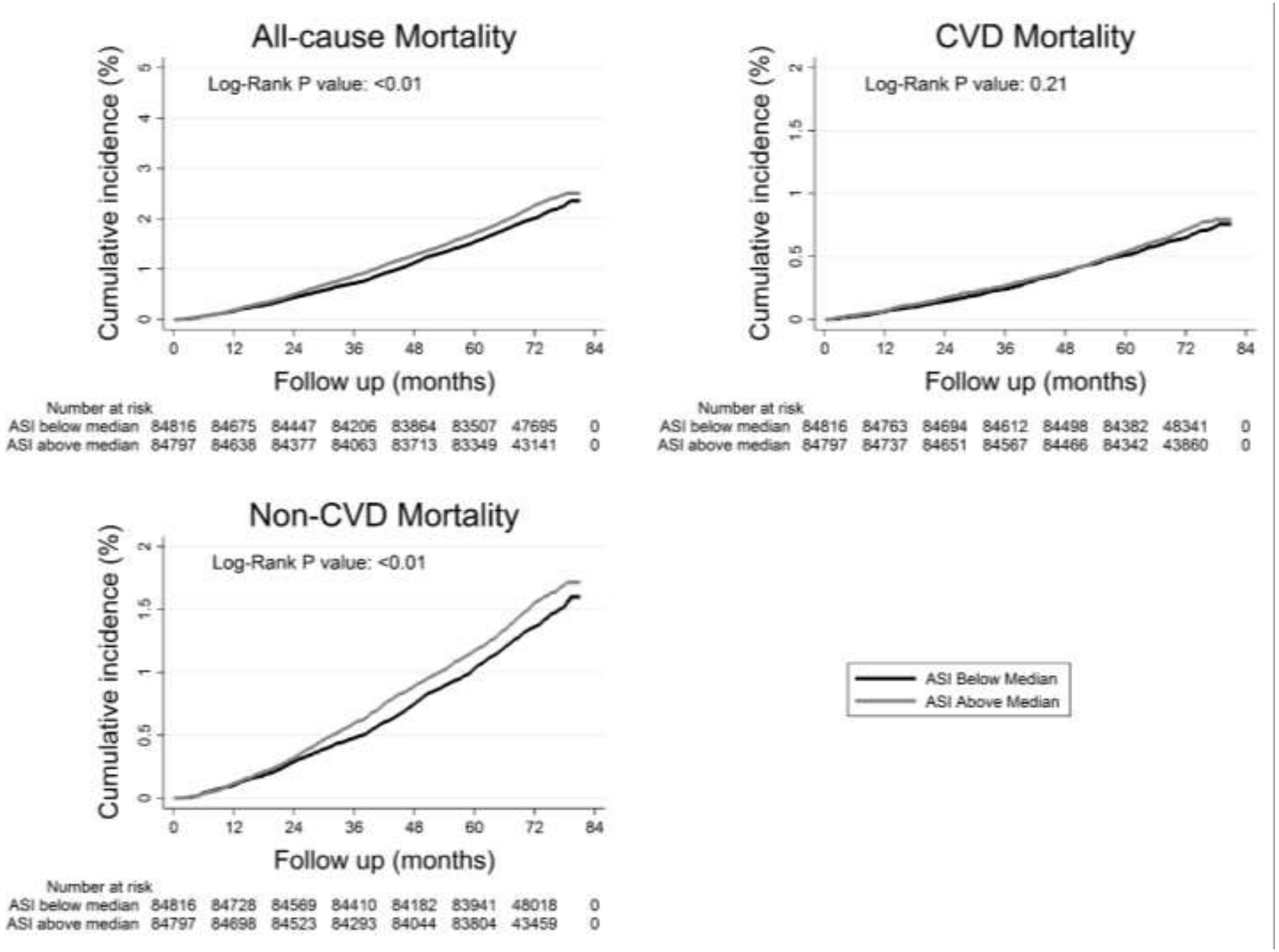

Figure S6. Shown are cumulative all-cause, CVD and non-CVD mortality in (%) divided by the median ASI. All curves were adjusted for age and sex. Log-Rank testing shows significant differences for all-cause and non-CVD, but not CVD mortality. Abbreviations: ASI, Arterial Stiffness Index; CVD, Cardiovascular Disease

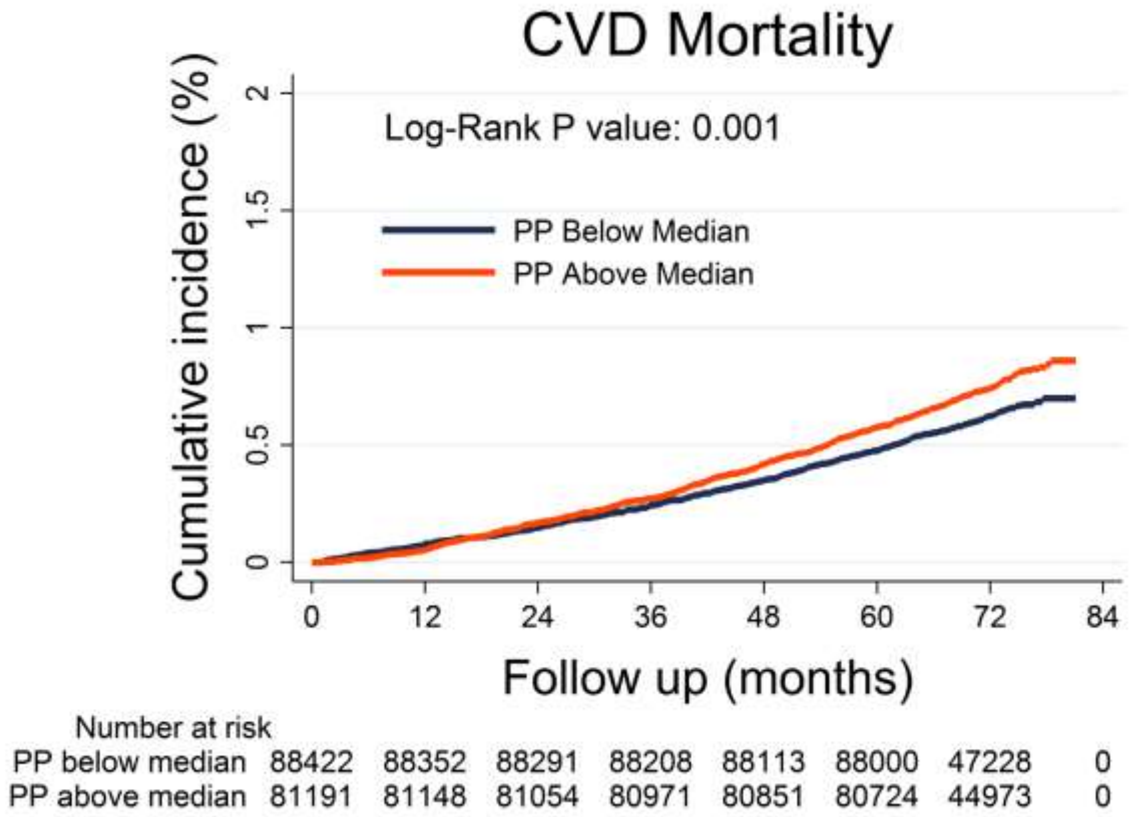

Figure S7. Shown is the cumulative CVD-mortality in (%) divided by the median PP. The curve was adjusted for age and sex. Log-Rank testing shows a significant difference. Abbreviations: PP, Pulse Pressure; CVD, Cardiovascular Disease
